# Characterization of raffinose metabolism genes uncovers a wild *Arachis* galactinol synthase conferring tolerance to abiotic stresses

**DOI:** 10.1038/s41598-020-72191-4

**Published:** 2020-09-17

**Authors:** Christina C. Vinson, Ana P. Z. Mota, Brenda N. Porto, Thais N. Oliveira, Iracyara Sampaio, Ana L. Lacerda, Etienne G. J. Danchin, Patricia M. Guimaraes, Thomas C. R. Williams, Ana C. M. Brasileiro

**Affiliations:** 1 EMBRAPA Recursos Genéticos e Biotecnologia. Parque Estação Biológica, Final W5 Norte, Brasília, DF CP 02372 Brazil; 2 Departamento de Botânica, Universidade de Brasília, Campus Darcy Ribeiro, Brasília, DF Brazil; 3 Université Côte d’Azur, INRAE, CNRS, ISA, Sophia-Antipolis, France

**Keywords:** Gene expression profiling, Transgenic plants

## Abstract

Raffinose family oligosaccharides (RFOs) are implicated in plant regulatory mechanisms of abiotic stresses tolerance and, despite their antinutritional proprieties in grain legumes, little information is available about the enzymes involved in RFO metabolism in Fabaceae species. In the present study, the systematic survey of legume proteins belonging to five key enzymes involved in the metabolism of RFOs (galactinol synthase, raffinose synthase, stachyose synthase, alpha-galactosidase, and beta-fructofuranosidase) identified 28 coding-genes in *Arachis duranensis* and 31 in *A. ipaënsis*. Their phylogenetic relationships, gene structures, protein domains, and chromosome distribution patterns were also determined. Based on the expression profiling of these genes under water deficit treatments, a galactinol synthase candidate gene (*AdGolS3*) was identified in *A. duranensis*. Transgenic *Arabidopsis* plants overexpressing *AdGolS3* exhibited increased levels of raffinose and reduced stress symptoms under drought, osmotic, and salt stresses. Metabolite and expression profiling suggested that *AdGolS3* overexpression was associated with fewer metabolic perturbations under drought stress, together with better protection against oxidative damage. Overall, this study enabled the identification of a promising *GolS* candidate gene for metabolic engineering of sugars to improve abiotic stress tolerance in crops, whilst also contributing to the understanding of RFO metabolism in legume species.

## Introduction

Persistent abiotic stress conditions, such as drought, salinity and extreme temperatures, are major environmental adverse factors that negatively affect the physiology and biochemistry of plants and limit crop production worldwide. To withstand the damaging effects of the intracellular water loss caused by the different types of abiotic stresses, plants have evolved a molecular arsenal for stress sensing, signaling, acclimation, and defense^1^. Complex molecular networks are thus activated to allow plants to cope with stressful conditions^2^, including modifications in plant metabolism to produce a series of low molecular weight compounds that accumulate in the cytosol or vacuole, such as soluble sugars, amines and amino acids^3^. These compounds participate in osmotic adjustment, stabilize cell components, provide membrane protection, and scavenge excess Reactive Oxygen Species (ROS)^4^.

The raffinose family oligosaccharides (RFOs), such as raffinose, stachyose and verbascose, have been intensively studied as osmoprotectants that accumulate in response to abiotic stress, representing a crucial element of plant defense mechanisms^3,5^. These oligosaccharides also have many other functions in plants, including protection of the embryo during maturation and desiccation of seeds, long-distance sugar transport in the phloem sap, storage of carbon, protection of the photosynthetic apparatus, mRNA export, and as signaling molecules in plant defense upon wounding or pathogen infection.

Biosynthesis of RFOs begins with the conversion of uridine diphosphate-galactose (UDP-Gal) and myo-inositol to galactinol catalyzed by galactinol synthase (GolS)^6^. GolS has a significant influence over RFO accumulation, as the first enzyme that commits carbon to RFO biosynthesis, and hence influences carbon partitioning between sucrose and RFOs. Raffinose, the first member of this series of RFOs, is synthesized by raffinose synthase (RS) through the transfer of a galactosyl moiety from galactinol to sucrose. The subsequent addition of another galactosyl residue to raffinose by stachyose synthase (STS) results in the production of stachyose^7^. Raffinose and stachyose can then be cleaved to produce a number of different oligosaccharides. The removal of the fructosyl unit of raffinose and stachyose by beta-fructofuranosidase (BFLUCT) leads to the formation of melibiose and manninotriose, respectively. Galactose can also be removed from RFOs through the action of alpha-galactosidase (AGAL).

Galactinol and RFOs are ubiquitous in plants and the genes coding for enzymes involved in their metabolism have been identified and characterized in some crop species, including *Zea mays*^8^, *Manihot esculenta*^9^, *Sesamum indicum*^10^, *Solanum lycopersicum*^11^ and *Malus* × *domestica*^12^. Whilst RFOs are major soluble sugars that are second only to sucrose in abundance in mature legume seeds^13^, very little is known about the genes involved in raffinose metabolism in Fabaceae species, with few examples described in *Cicer arietinum* and *Glycine max*^14,15^. RFOs are indeed antinutritional and undesirable compounds of legume seeds and the development of cultivars with reduced levels of raffinose and stachyose has been the focus of breeding programs for improving nutritional quality in many grain legumes^13,16,17^. In peanut (*Arachis hypogaea*), the second most cultivated grain legume in the world after soybean, seeds also accumulate high amounts of indigestible RFOs, which limits its consumption^13,17^.

Over the past years, we have exploited the peanut wild relatives gene pool as a source of valuable traits for peanut breeding, such as resistance/tolerance to abiotic and biotic stresses^18^. Progress in functional genomics of wild *Arachis* led to the discovery of many genes and proteins involved in water deficit responses in the drought-tolerant *A. duranensis* and in the drought-sensitive *A. stenosperma*, including genes involved in RFO metabolism^19–24^. Furthermore, the availability of the complete genome sequences of the wild ancestral species of peanut, *A. duranensis* and *A. ipaënsis* (https://www.peanutbase.org/), has enabled genome-wide analysis and contributed to new insights into the molecular function and evolution of gene families in the genus *Arachis*^25^. However, despite the important role of peanut, and other legumes, as protein sources for human and animal consumption, a detailed study of the genomic organization of RFO metabolic genes is still missing.

In the present study, a genome-wide analysis identified 28 genes in *A. duranensis* and 31 in *A. ipaënsis* as coding for five key enzymes of the RFO biosynthesis, accumulation, and catabolism (GolS, RS, STS, AGAL and BFLUCT). Phylogenetic relationships, gene and protein structures, and the chromosome distribution patterns of these genes were also characterized. Additionally, analysis of transcriptome data of different wild *Arachis* species submitted to water deficit treatments identified the *AdGolS3* gene as a candidate gene for drought tolerance. The effects of *AdGolS3* overexpression in transgenic *Arabidopsis* plants and its putative role in abiotic stress responses were investigated. Our findings indicated *AdGolS3* may act preventively to protect plant cells against oxidative damage and therefore contribute to drought tolerance.

## Methods

### Construction of profile hidden Markov models and scans of Fabaceae predicted proteomes

The protein sequences of five key enzymes involved in plant raffinose metabolism were downloaded from Carbohydrate-Active enZyme database (CAZy; https://www.cazy.org/;^26^). In CAZy, only the proteins whose biochemical activity has been experimentally confirmed are assigned an Enzyme Commission (EC) number. We thus retrieved from legumes the glycosyltransferase family 8 (GT8) galactinol synthase (GolS; EC 2.4.1.123) and the glycoside hydrolases: family 36 (GH36) raffinose synthase (RS; EC 2.4.1.82) and stachyose synthase (STS; EC 2.4.1.67); family 27 (GH27) alpha-galactosidase (AGAL; EC 3.2.1.22); and family 32 (GH32) beta-fructofuranosidase (BFLUCT; EC 3.2.1.26). For each selected CAZyme family, protein sequences found in Fabaceae species were aligned using MAFFT software^27^ and trimmed with trimAL software, to eliminate regions with more than 10% gaps^28^. For each of the five enzymes, a profile hidden Markov model (profile-HMM) was built using the hmmbuild function from HMMER suite v3.1 (https://hmmer.org/;^29^), with the aligned and trimmed protein sequences as input.

The predicted proteomes of *A. duranensis* and *A. ipaënsis*^25^ were retrieved from PeanutBase (https://peanutbase.org/) and the protein sets of the remaining Fabaceae species were downloaded from the UniProt database (https://www.uniprot.org/taxonomy/fabaceae/). The five HMM profiles (for GolS; RS; STS; AGAL and BFLUCT) were then used as queries against this set of proteomes to find the matching proteins, using the hmmsearch function from HMMER v3.1 (https://hmmer.org/). Only the protein sequences with a full sequence score > 100 were retained and considered for further analyses. The CD-HIT software (https://weizhongli-lab.org/cd-hit/;^30^) was used to eliminate the redundant proteins, with a cut-off sequence identity of 90%. Details of the methods used for final multiple sequence alignments and phylogenetic analysis as well as the analysis of gene/protein structures and genomic distribution in *Arachis* spp. are given in the Supplementary Methods.

### In silico expression profiling of *A. duranensis* genes

Illumina RNA-seq data previously obtained by our group were used to determine the in silico expression profiles of GolS, RS, STS, AGAL and BFLUCT genes in *Arachis* spp. This data comprises: (1) Transcripts expressed in roots of *A. duranensis* and *A. stenosperma* plants submitted to dehydration treatment, by the withdrawal of hydroponic nutrient solution from 25 to 150 min^21^ and pooled in equal amounts, a treatment which we previously showed to induce major alterations in the transcriptome of these species. (2) Transcripts expressed in *A. duranensis* and *A. stenosperma* plants subjected to a decreased in soil availability with withholding of irrigation for four days, a treatment which induced proteomic and transcriptomic alterations^24^. The differential expression values (log2 of fold-change) between stressed and control samples in *A. duranensis* and *A. stenosperma* roots were plotted in a heatmap graph using the heatmap2 from ggplot R package, as previously described^23^.

### *Arabidopsis thaliana* lines overexpressing *AdGolS3* gene

The coding sequence of the *AdGolS3* gene was identified by the alignment of the Aradu.ZK8VV gene model (https://peanutbase.org) with the four best BLASTn hits of *A. duranensis* at NCBI (https://www.ncbi.nlm.nih.gov). The obtained *AdGolS3* consensus sequence (981 bp) was synthetized and cloned under the control of the *A. thaliana* actin 2 promoter in the binary vector pPZP_201BK_EGFP^31^ by Epoch Life Science Inc. (TX, USA). The resulting vector, pPZP-AdGolS3, was transferred to the *Agrobacterium* disarmed strain ‘GV3101’ and the transformed colonies selected by PCR with specific eGFP and AdGolS3 primers (Table S1), using standard protocols.

Wild-type (WT) *A. thaliana* plants (ecotype Columbia; Col-0) were transformed with the GV3101-pPZP-AdGolS3 *Agrobacterium* strain by the floral dip immersion method^32^. The eGFP-positive and hygromycin-resistant T0 transformants were grown in a controlled growth chamber (21 °C with a 12 h photoperiod and light intensity of 120 µmols.m^−2^.s^−1^) to obtain transgenic *AdGolS3* overexpressing (OE) lines, as described previously^22^. T1 seeds of each transgenic line obtained by self-pollination of T0 plants were germinated on hygromycin selective medium and T1 plants grown to maturity. Self-pollinated T2 seeds derived from each T1 plant were maintained separate and 24 T2 seeds tested for homozygosity through germination on hygromycin selective medium. If all T2 seeds germinated in hygromycin selective medium, we considered that they derived from a T1 plant homozygote producing T2 homozygous seeds. All the subsequent stress treatments and analyses were conducted with homozygous *AdGolS3*-OE plants of the T2 generation.

### Transgene expression and sugar content in *AdGolS3*-OE lines

The expression of *AdGolS3* transgene in 13 independent *Arabidopsis* OE lines was confirmed through qRT-PCR analysis as described below (2.10), using specific AdGolS3 primers (Table S1). The content of four sugars (glucose, fructose, sucrose and raffinose) was determined in leaves of one-month-old WT and transgenic plants (five individuals per genotype) as described previously^33^. Sugars were extracted using 80% (v/v) ethanol at 80 °C, and the extracts dried and resuspended in water for analysis. Samples were analyzed using a High Performance Anion Exchange (HPAE) chromatography system (Dionex, ICS 3,000, Sunnyvale, CA, USA) equipped with a pulsed amperometric detector and Carbopak PA-10 column. Sugars were separated using an isocratic method with 52 mM NaOH and a column flow of 0.2 mL.min^−1^ over 35 min and quantified using standard curves.

### Dry-down assay

Based on the HPAE analysis, three *AdGolS3-*OE lines (GolS17, GolS20 and GolS22) that showed significantly higher levels of raffinose compared to WT were selected for the subsequent abiotic stress assays. Seeds from WT and transgenic plants were sown in 250 mL pots containing the same amount of substrate (Carolina Soil, CSC, Brazil) and maintained under the growth conditions described above. The dry-down assay started when the plants were 30 days old and lasted for 20 days. Plants were divided into three treatments: (1) Control (CTR) group maintained under irrigated conditions, i.e., around 70% of field capacity (FC); (2) Stressed (STR) group where irrigation was suspended; and (3) Rehydrated (REH) group where STR plants were irrigated 24 h before collection. CTR, STR and REH treatments started at the same time and were carried out in parallel, each group with its own set of plants. At the end of the assay, ten individuals from each treatment (CTR, STR and REH) were collected (at 9 am) for each genotype, weighed and stored at -80 °C for later biochemical and molecular analyses.

The leaf disc submersion methods were used for the determination of the relative water content (RWC) and electrolyte leakage (EL), as described previously^34,35^. For RWC and EL measurements, three leaf discs of 0.4 cm^2^ were used per individual for each treatment (CTR, STR and REH).

### Soluble sugar analysis and metabolic profiling

Soluble sugars (glucose, sucrose and raffinose) were separated and quantified in the three selected *AdGolS3-*OE lines and in WT plants under CTR, STR and REH conditions using the HPAE chromatography system, as described above. Metabolic profiling of these four genotypes was carried out according to^36^. Samples of lyophilized tissue were extracted using the methanol:chloroform:water method with ribitol as an internal standard. Aliquots of the polar phase were dried and derivatized using methyoxyamine hydrochloride in pyridine followed by MSTFA. Samples were analyzed using an Agilent 7820A GC coupled to an Agilent 5,975 MSD equipped with a 30 m HP5-ms column. Metabolites were identified by comparison with a custom mass spectral library and chromatograms were aligned using MetaAlign^37^.

### NaCl and PEG treatments

Based on the results of the dry-down experiment, the GolS22 OE line was selected for evaluation of its performance in response to two additional abiotic stress treatments: NaCl (salt stress) and polyethylene glycol (PEG; osmotic stress). WT and GolS22 plants were grown as described above for 30 days then divided between three groups: (1) Control group maintained under irrigated conditions (maintained at 70% FC); (2) Salt stressed group irrigated with a 150 mM NaCl solution instead of water; and (3) Osmotic stressed group irrigated with a PEG 6,000 20% (w/v) solution instead of water. Plants from each group were maintained under these conditions for 15 days. Ten individuals from each treatment were collected (at 9 am) per genotype, weighed and analyzed for RWC and EL, as described above.

### qRT-PCR analysis

The relative expression of the *AdGolS3* transgene and of a subset of *Arabidopsis* genes was determined in WT plants and OE lines by qRT-PCR analysis, as previously described^22^. This gene subset comprises five *Arabidopsis* genes selected based on their putative interaction with *AtGolS2* in *Arabidopsis*, as predicted by geneMANIA^38^. It includes (Table S1): Glutathione S-transferase (*AtGSTU24*), Stress‐Associated Protein (*AtSAP13*), Ascorbate Peroxidase (*AtAPX1*), Peroxisomal Catalase (*AtCAT2*) and Alpha amylase family protein (*AtEMB2729*). *AtGolS2* is the orthologous of the *AdGolS3* gene in *Arabidopsis* and was included in the qRT-PCR analysis. Specific primers were designed (Table S1) using the software Primer3Plus, following the parameters described previously^39^. The qRT-PCR reactions were performed on a StepOne Plus Real-Time PCR System (Applied Biosystems, Foster City, USA) in technical triplicates for each sample, using No Template (NTC) and No Amplification (NAC) samples as negative controls. The relative quantification (RQ) of mRNA levels was normalized with *AtACT2* and *AtEF-1α* reference genes (Table S1).

## Results

### Genome-wide identification of wild *Arachis* genes involved in RFO metabolism

#### Galactinol synthase (GolS)

We identified six plant proteins with experimentally verified biochemical function in the CAZy database as GolS proteins that shared the common conserved PFAM domain (PF01501) of the GT8 family. The two belonging to the Fabaceae family (GLYMA Q7XZ08 and MEDSA Q84MZ5) were then used as references for HMM profile construction.

Using this HMM profile, 30 proteins from 11 Fabaceae species were identified in UniProt as putatively belonging to the GolS family (Table S2), together with five proteins each for *A. duranensis* and *A. ipaënsis* genomes. These 10 *Arachis* putative GolS proteins ranged from 311 to 341 amino acids in length (average of 328) without signal peptides (Table 1). Phylogenetic analysis performed with the 40 putative GolS proteins revealed two distinct clusters: cluster 1, with 15 proteins, and cluster 2 with 25 (Fig. S1a). *Arachis* GolS proteins had representatives in both clusters, with six proteins in cluster 1 and four in cluster 2 (Fig. S1a).

**Table 1:** Raffinose metabolism genes in *Arachis* spp.

Family	Name	Species	Gene name	Chromosome	Start	End	Duplication type	Signal peptide
GolS	Aradu.EQ3HU	*Arachis duranensis*	*AdGolS1*	Aradu.A09	22,459,116	22,460,518	WGD	NO
	Aradu.P39C1	*Arachis duranensis*	*AdGolS2*	Aradu.A06	6,944,440	6,947,461	WGD	NO
	Aradu.ZK8VV	*Arachis duranensis*	*AdGolS3*	Aradu.A06	5,706,973	5,708,738	WGD	NO
	Aradu.Z0EIQ	*Arachis duranensis*	*AdGolS4*	Aradu.A09	18,124,933	18,127,295	WGD	NO
	Aradu.9K3NU	*Arachis duranensis*	*AdGolS5*	Aradu.A09	18,134,426	18,138,923	Tandem	NO
	Araip.L3NWH	*Arachis ipaënsis*	*AiGolS1*	Araip.B09	28,388,070	28,389,727	WGD	NO
	Araip.M48BY	*Arachis ipaënsis*	*AiGolS2*	Araip.B06	9,687,845	9,689,970	WGD	NO
	Araip.NEM14	*Arachis ipaënsis*	*AiGolS3*	Araip.B06	11,622,775	11,624,454	WGD	NO
	Araip.SRA93	*Arachis ipaënsis*	*AiGolS4*	Araip.B09	23,495,394	23,497,841	WGD	NO
	Araip.40N3F	*Arachis ipaënsis*	*AiGolS5*	Araip.B09	23,506,965	23,510,107	Proximal	NO
RS	Aradu.HC115	*Arachis duranensis*	*AdRS1*	Aradu.A06	11,295,903	11,300,129	Dispersed	NO
	Aradu.G4PX6	*Arachis duranensis*	*AdRS2*	Aradu.A07	1,275,877	1,279,624	Dispersed	NO
	Aradu.BR5RX	*Arachis duranensis*	*AdRS3*	Aradu.A01	93,996,626	93,999,785	Dispersed	NO
	Aradu.J3GMZ	*Arachis duranensis*	*AdRS4*	Aradu.A03	39,901,809	39,907,567	Dispersed	NO
	Aradu.F2QXB	*Arachis duranensis*	*AdRS5*	Aradu.A05	86,738,383	86,738,756	Dispersed	NO
	Aradu.Q8JL3	*Arachis duranensis*	*AdRS6*	Aradu.A03	44,328,240	44,341,051	Dispersed	NO
	Aradu.YX91W	*Arachis duranensis*	*AdRS7*	Aradu.A03	40,458,135	40,464,544	Dispersed	NO
	Araip.KNT6A	*Arachis ipaënsis*	*AiRS1*	Araip.B07	972,692	976,495	Dispersed	NO
	Araip.57L7Q	*Arachis ipaënsis*	*AiRS2*	Araip.B01	133,866,829	133,869,985	Dispersed	NO
	Araip.5E5Q0	*Arachis ipaënsis*	*AiRS3*	Araip.B03	1,783,130	1,791,977	WGD	NO
	Araip.EW2ZU	*Arachis ipaënsis*	*AiRS4*	Araip.B05	141,696,277	141,702,037	Dispersed	NO
	Araip.LBU7J	*Arachis ipaënsis*	*AiRS5*	Araip.B03	46,915,043	46,927,158	Dispersed	NO
	Araip.87RQD	*Arachis ipaënsis*	*AiRS6*	Araip.B07	160,342	163,931	Dispersed	NO
STS	Aradu.K6F4E	*Arachis duranensis*	*AdSTS1*	Aradu.A06	7,161,722	7,173,062	Dispersed	NO
	Araip.371ZH	*Arachis ipaënsis*	*AiSTS1*	Araip.B06	9,408,043	9,411,003	Tandem	NO
	Araip.KD23G	*Arachis ipaënsis*	*AiSTS2*	Araip.B06	9,392,860	9,396,289	Tandem	NO
	Araip.V1U21	*Arachis ipaënsis*	*AiSTS3*	Araip.B03	41,723,282	41,724,718	Dispersed	NO
AGAL	Aradu.XWB1G	*Arachis duranensis*	*AdAGAL1*	Aradu.A10	16,753,344	16,757,269	WGD	YES
	Aradu.8WX2Z	*Arachis duranensis*	*AdAGAL2*	Aradu.A04	103,979,917	103,983,868	WGD	YES
	Aradu.47BJJ	*Arachis duranensis*	*AdAGAL3*	Aradu.A10	70,967,137	70,973,272	Dispersed	NO
	Aradu.01PEQ	*Arachis duranensis*	*AdAGAL4*	Aradu.A05	84,051,791	84,059,234	Dispersed	NO
	Araip.WG870	*Arachis ipaënsis*	*AiAGAL1*	Araip.B10	23,922,271	23,926,161	Dispersed	YES
	Araip.L2HQR	*Arachis ipaënsis*	*AiAGAL2*	Araip.B04	112,413,967	112,417,806	Dispersed	YES
	Araip.EXU6F	*Arachis ipaënsis*	*AiAGAL3*	Araip.B10	98,873,071	98,879,240	Dispersed	NO
	Araip.VT0TG	*Arachis ipaënsis*	*AiAGAL4*	Araip.B05	144,126,670	144,129,965	Dispersed	NO
BFLUCT	Aradu.8MB2V	*Arachis duranensis*	*AdBFLUCT1*	Aradu.A09	6,611,685	6,619,003	WGD	YES
	Aradu.27C7S	*Arachis duranensis*	*AdBFLUCT2*	Aradu.A08	22,961,400	22,964,409	WGD	YES
	Aradu.23E3L	*Arachis duranensis*	*AdBFLUCT3*	Aradu.A09	7,652,756	7,660,363	Dispersed	NO
	Aradu.W64DR	*Arachis duranensis*	*AdBFLUCT4*	Aradu.A08	16,812,196	16,815,218	WGD	YES
	Aradu.ET296	*Arachis duranensis*	*AdBFLUCT5*	Aradu.A06	108,864,036	108,867,587	WGD	YES
	Aradu.T5FHF	*Arachis duranensis*	*AdBFLUCT6*	Aradu.A06	108,878,603	108,881,468	Proximal	NO
	Aradu.YZR0Z	*Arachis duranensis*	*AdBFLUCT7*	Aradu.A02	92,641,782	92,647,101	Dispersed	NO
	Aradu.RZZ9E	*Arachis duranensis*	*AdBFLUCT8*	Aradu.A06	70,531,598	70,535,609	Dispersed	YES
	Aradu.NB8XZ	*Arachis duranensis*	*AdBFLUCT9*	Aradu.A03	11,428,507	11,436,420	Dispersed	NO
	Aradu.1B80H	*Arachis duranensis*	*AdBFLUCT10*	Aradu.A01	41,923,639	41,930,127	WGD	NO
	Aradu.7N2H0	*Arachis duranensis*	*AdBFLUCT11*	Aradu.A05	13,499,316	13,503,397	WGD	NO
	Araip.C8IBG	*Arachis ipaënsis*	*AiBFLUCT1*	Araip.B09	8,212,457	8,219,686	WGD	YES
	Araip.XJD82	*Arachis ipaënsis*	*AiBFLUCT2*	Araip.B08	1,317,638	1,321,036	WGD	YES
	Araip.DD0BF	*Arachis ipaënsis*	*AiBFLUCT3*	Araip.B09	9,475,352	9,484,989	Dispersed	NO
	Araip.ET6IJ	*Arachis ipaënsis*	*AiBFLUCT4*	Araip.B07	124,769,441	124,772,982	WGD	NO
	Araip.9LD3P	*Arachis ipaënsis*	*AiBFLUCT5*	Araip.B06	133,132,276	133,136,581	WGD	YES
	Araip.HL8V4	*Arachis ipaënsis*	*AiBFLUCT6*	Araip.B06	133,147,400	133,150,417	Proximal	NO
	Araip.0311L	*Arachis ipaënsis*	*AiBFLUCT7*	Araip.B02	107,331,957	107,337,444	Dispersed	NO
	Araip.6800 W	*Arachis ipaënsis*	*AiBFLUCT8*	Araip.B06	88,870,223	88,874,024	Dispersed	YES
	Araip.571BV	*Arachis ipaënsis*	*AiBFLUCT9*	Araip.B08	8,176,140	8,183,448	Proximal	NO
	Araip.Q5XJH	*Arachis ipaënsis*	*AiBFLUCT10*	Araip.B08	8,243,124	8,244,014	Proximal	NO
	Araip.KLH8I	*Arachis ipaënsis*	*AiBFLUCT11*	Araip.B03	14,010,059	14,019,188	Dispersed	NO
	Araip.RKE1H	*Arachis ipaënsis*	*AiBFLUCT12*	Araip.B01	49,729,438	49,734,903	Dispersed	NO
	Araip.28YBL	*Arachis ipaënsis*	*AiBFLUCT13*	Araip.B05	14,535,718	14,540,357	Dispersed	NO

Genes coding for galactinol synthase (GolS), raffinose synthase (RS), stachyose synthase (STS), alpha-galactosidase (AGAL) and beta-fructofuranosidase (BFLUCT) identified in *Arachis duranensis* and *Arachis ipaënsis.*

Phylogenetic clustering was associated with the number of exons, with all genes coding the six proteins from cluster 1 consistently having three exons and those from cluster 2 having three to five exons (Fig. S1b). Three highly-conserved protein motifs were identified in the in the *Arachis* GolS family (Fig. S1c).

#### Raffinose synthase (RS) and stachyose synthase (STS)

Although the RS and the STS families have distinct EC codes, they are characterized by the presence of the same conserved GH36 family domain (PFAM domain PF05691). We found two plant proteins functionally characterized as belonging to the RS family and only one from the STS family in the CAZy database that were then used for the construction of separate RS and STS HMM profiles.

In total, 62 putative RS proteins were identified in 11 Fabaceae species in UniProt (Table S2). Another seven RS proteins were found in *A. duranensis* and six in *A. ipaënsis*, with an average length of 773 amino acids (ranging between 546 and 1,035) and lack of signal peptides (Table 1). Phylogenetic analysis of the 75 putative Fabaceae RS separated these proteins into seven distinct clusters (Fig. S2a). The *Arachis* RS proteins were evenly distributed among six clusters, with one protein per cluster, except for cluster 1, with two *A. duranensis* proteins (AdRS1 and AdRS2). As for GolS, RS protein clustering is related to its gene organization in *Arachis*, with genes belonging to the same cluster presenting a similar number of exons (Fig. S2b), which ranges from three exons in cluster 2 to more than 12 exons in clusters 1 and 3. The analysis of proteins sequences showed the presence of three conserved motifs in the 13 *Arachis* RS (Fig. S2c).

Fourteen putative STS proteins were retrieved from nine Fabaceae species (Table S2), including three from *A. ipaënsis* and only one from *A. duranensis.* These four putative STS *Arachis* proteins ranged from 359 to 1,696 amino acids in length (average of 945), without signal peptides (Table 1). Phylogenetic analysis revealed a highly conserved STS family in Fabaceae, forming two clusters: the first one with all proteins exclusive to the genus *Arachis* (AdSTS1, AiSTS1, AiSTS2 and AiSTS3) and the second with 10 proteins from other Fabaceae species (Fig. S3a). Exceptionally, no relationship was observed between this phylogenetic clustering and the intron/exon organization of the STS *Arachis* genes (Fig. S3b). The sequences of the four *Arachis* STS proteins showed at least three conserved motifs (Fig. S3c).

#### Alpha-galactosidase (AGAL)

AGAL is part of the GH27 enzyme family and is characterized by the presence of the conserved PFAM domain of melibiase_2 (PF16499). We retrieved three functionally characterized AGAL plant proteins from the CAZy database that were used to construct the HMM profile.

This profile revealed 56 putative AGAL proteins from 14 Fabaceae species (Table S2), from which four proteins belonged to *A. duranensis* and four to *A. ipaënsis*, with an average length of 374 amino acids, ranging from 178 to 437. Phylogenetic analysis divided these 56 putative AGAL proteins into three clusters (Fig. S4a). Clusters 2 and 3 each contain a single representative from *A. duranensis* and *A. ipaënsis,* whereas cluster 1 has two representatives from each species. Interestingly, the two *A. duranensis* (AdAGAL1 and AdAGAL2) and *A. ipaënsis* (AdAGAL1 and AdAGAL2) AGAL proteins that contained signal peptides shared the same cluster 1 (Table 1 and Fig. S4a). The *Arachis* proteins within the same cluster showed a similar intron/exon structure, except for those in cluster 3, where the *A. duranensis AdAGAL4* gene had 15 exons while *A. ipaënsis AiAGAL4* contained only seven (Fig. S4b). The protein sequence analysis showed that all eight *Arachis* AGAL proteins share at least three conserved common motifs (Fig. S4c).

#### Beta-fructofuranosidase (BFLUCT)

The BFLUCT enzyme family belongs to CAZy family GH32 and is also defined by the presence of two glycosyl hydrolase PFAM domains: Glyco_Hydro32N (PF00251) and Glyco_Hydro32C (PF08244). In the CAZy database, 78 plant proteins were functionally characterized as BFLUCT and we used the 11 Fabaceae proteins as references for the construction of an HMM profile.

A total of 114 putative BFLUCT proteins was retrieved from 13 Fabaceae species (Table S2), besides 11 proteins in *A. duranensis* and 13 in *A. ipaënsis*, with an average length of 552 amino acids, ranging from 140 to 676 (Table 1). Phylogenetic analysis produced five clusters, from which, four contained at least one representative of each *Arachis* species (Fig. S5a). Five *A. duranensis* and four *A. ipaënsis* BFLUCT proteins harbored signal peptides, but, unlike the AGAL proteins, these proteins did not share the same clusters (Fig. S5a). In general, the intron/exon organization of genes belonging to the same protein cluster was similar (Fig. S5b). Three protein motifs were identified and conserved in all *Arachis* BFLUCT, except for *AdBFLUCT6* (Fig. S5c).

### Genomic distribution and duplication patterns of RFO metabolism genes in wild *Arachis*

**Figure 1:**
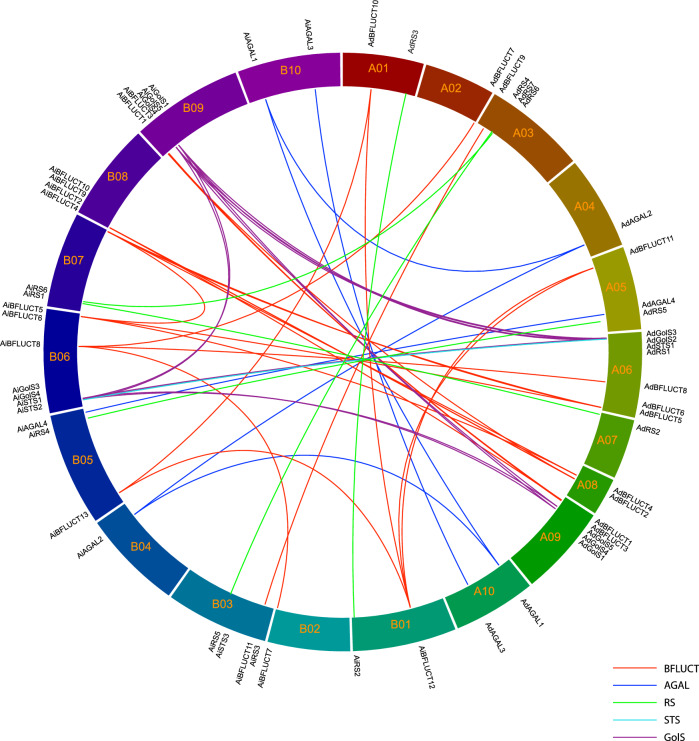
Genomic distribution of RFO metabolism *Arachis* genes. Distribution of galactinol synthase (GolS), raffinose synthase (RS), stachyose synthase (STS), alpha-galactosidase (AGAL) and beta-fructofuranosidase (BFLUCT) genes in the ten chromosomes of *Arachis duranensis* (A01–A10) and *A. ipaënsis* (B01–B10). Synteny between the two genomes is represented by lines. GolS (purple), STS (yellow), RS (green), AGAL (blue) and BFLUCT (red). The figure was generated by Circa software (https://omgenomics.com).

Genome-wide analysis of the two wild *Arachis* species with genome sequences so far available identified 28 RFO metabolism genes in *A. duranensis* and 31 in *A. ipaënsis*. In both species, these genes were unevenly distributed in the ten chromosomes regardless of the enzyme family (Fig. 1). The majority of the 59 genes were restricted to the distal chromosomal regions, in accordance with previous studies showing the gene-rich characteristic of these hot recombination hotspot regions in wild *Arachis* genomes^23,25,40^.

All the genes identified as involved in raffinose metabolism were duplicated in both *Arachis* species. The majority of gene copies (50.8%) resulted from dispersed duplications, 35.6% originated from whole-genome (WGD)/segmental duplication, 8.5% from proximal duplication and 5.1% from tandem duplications (Table 1). In the GolS family, specifically, the gene copies resulted mostly from WGD/segmental duplications (80%), while in the RS and AGAL families, the dispersed gene duplications represented the majority (92.3% and 75%, respectively; Table 1). Expansion of STS family genes similarly resulted from tandem and dispersed duplications (50% each) and the BFLUCT family from WGD/segmental and dispersed duplications (41.7% each) (Table 1).

### Expression profiling of *Arachis* RFO metabolism genes in response to water deficit

**Figure 2:**
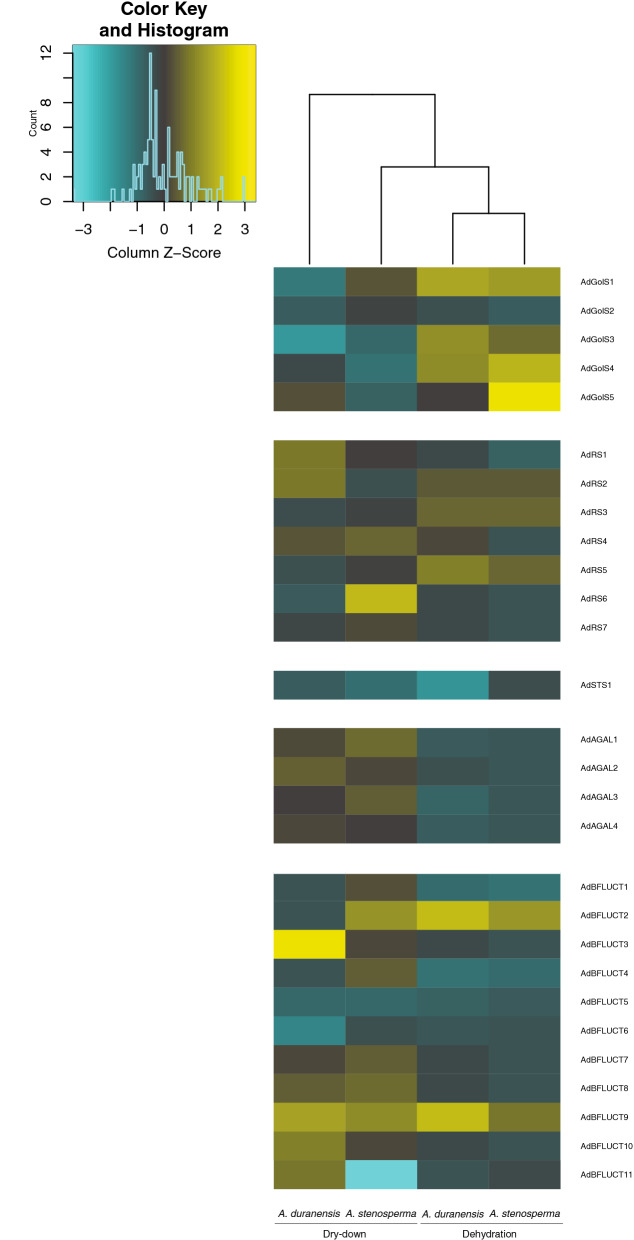
Expression profiling of RFO metabolism *Arachis* genes. Heatmap of the 28 transcripts identified as involved in RFO metabolism (GolS; RS; STS; AGAL and BFLUCT) in *Arachis* spp. Expression patterns (log2-based values from RNA-seq data) of *Arachis duranensis* genes and *A. stenosperma* orthologs were determined in plants submitted to drought (dry-down) imposed by the gradual decrease in soil water availability and to dehydration by withdrawal of hydroponic nutrient solution. The heatmap was generated by ggplot2 version 3.1 in Rstudio.

The expression patterns of the 28 *A. duranensis* RFO metabolism genes in response to two types of drought imposition (dehydration and dry-down) were analyzed using our previous transcriptome data obtained from the drought-tolerant accession K7988 of *A. duranensis* and the drought-sensitive accession V10309 of *A. stenosperma*^21^. This analysis revealed that the expression of most of the genes involved in RFO metabolism were modulated in response to water deficit, with distinct patterns and expression levels depending on the type of stress imposed and, to a lesser extent, on the *Arachis* genotype (Fig. 2).

The *Arachis* RS genes exhibited small variations in their expression levels in response to the two types of drought imposition in both genotypes. The exception was *AdRS6* with an high upregulation (fold change > 3) under dry-down in *A. stenosperma* (Fig. 2). Concerning the single representative of the STS family, *AdSTS1* was moderately downregulated in both *Arachis* genotypes in response to the two stresses.

The expression profile of the four representatives of the AGAL family, the initial enzyme responsible for raffinose breakdown, was different in the two treatments, regardless of the *Arachis* genotype (Fig. 2). Under dehydration stress, AGAL genes exhibited an overall downregulation pattern, with low expression levels, whereas under the dry-down stress, they did not seem to be modulated. Likewise, most of the 11 *A. duranensis* BFLUCT genes were downregulated in response to the dehydration treatment whilst only being slightly affected by dry-down (Fig. 2). The exception was *AdBFLUCT3*, which was strongly induced (fold change > 10) in *A. duranensis* plants submitted to dry-down.

Genes coding for the five GolS showed contrasting expression behaviors in response to the two types of drought imposition, regardless of the *Arachis* genotype, with a general upregulation under dehydration and downregulation under dry-down (Fig. 2). This was especially evident for *AdGolS4* and *AdGolS5*, which exhibited strong upregulation (fold change > 4) in response to dehydration. *AdGolS2* was the only GolS gene downregulated in response to dehydration.

However, *AdGolS3* drew particular attention as it exhibited the greatest difference in expression between the dehydration (upregulation of 3.15-fold) and dry-down (downregulation of − 8.16-fold) treatments in the drought-tolerant *A. duranensis* (Fig. 2). Conversely, in the more drought-sensitive *A. stenosperma*, the difference in the expression magnitude between dehydration (upregulation of 2.19-fold) and the dry-down (downregulation of − 1.75-fold) was much smaller (Fig. 2). *AdGolS3* is also the orthologue of *Arabidopsis AtGolS2*, which is known to be responsive to diverse abiotic stresses, and confers enhanced drought tolerance when overexpressed in transgenic plants^41^. Given its differential regulation in drought tolerant and sensitive *Arachis* species, and orthologous relationship to *AtGolS2*, we therefore selected *AdGolS3* for further *in planta* functional studies, to better understand the role of *GolS* genes in the molecular response underlying the process of water loss in wild *Arachis*.

### Screening of *A. thaliana* lines overexpressing* AdGolS3*

The *AdGolS3* coding sequence was predicted by the alignment of five sequences: Aradu.ZK8VV (https://peanutbase.org); GW944818.1; XM_016113210.2; HP005973.1 TSA and GW952716.1 (https://www.ncbi.nlm.nih.gov). The consensus sequence of 981 bp was cloned into pPZP-AdGolS3 and used to produce T0 primary *Arabidopsis* transformants. A total of 13 independent homozygous OE lines at T2 generation were obtained and *AdGolS3* overexpression was confirmed by qRT-PCR analysis in all of these OE lines, with the expression levels relative to the two reference genes varying among individual lines (Fig. S6). *AdGolS3* expression was not detected in WT plants.

Given the putative involvement of *AdGolS3* in the synthesis of raffinose series sugars, the leaf sugar content in the 13 OE lines was also analyzed and compared to WT plants. Leaves from four OE lines (GolS10; GolS17; GolS20 and GolS22) showed a significantly higher level of raffinose compared to WT plants, whereas four (GolS2; GolS4; GolS6, and GolS8) unexpectedly had a significant decrease (p < 0.05, Fig. S7). There was no clear relationship between the transgene expression levels (Fig. S6) and raffinose contents (Fig. S7) in the 13 OE lines, consistent with the complex relationship between transcript abundance and accumulation of metabolites that lie downstream of the encoded enzyme. Overall, the content of other sugars also involved in RFO metabolism (glucose, fructose and sucrose) was not affected by *AdGolS3* overexpression (Fig. S7). The exception was the GolS20 OE line, which presented an indirect, and specific, effect in its overall sugar content, with a significant increase in the concentration of all four sugars (glucose, fructose, sucrose and raffinose) compared to WT (Fig. S7). Interestingly, this OE line showed one of the lowest levels of ectopic *AdGolS3* expression (Fig. S6). Based on these findings, and given previous reports of correlations between accumulation of RFOs and tolerance to stress^3,5^, the three promising OE lines that showed the highest levels of raffinose accumulation with variable levels of transgene expression (GolS17, GolS20 and GolS22) were selected for further stress assays and physiological and biochemical analyses.

### Analysis of *Arabidopsis* plants overexpressing *AdGolS3*

#### Plant growth, relative water content (RWC) and electrolyte leakage (EL)

**Figure 3:**
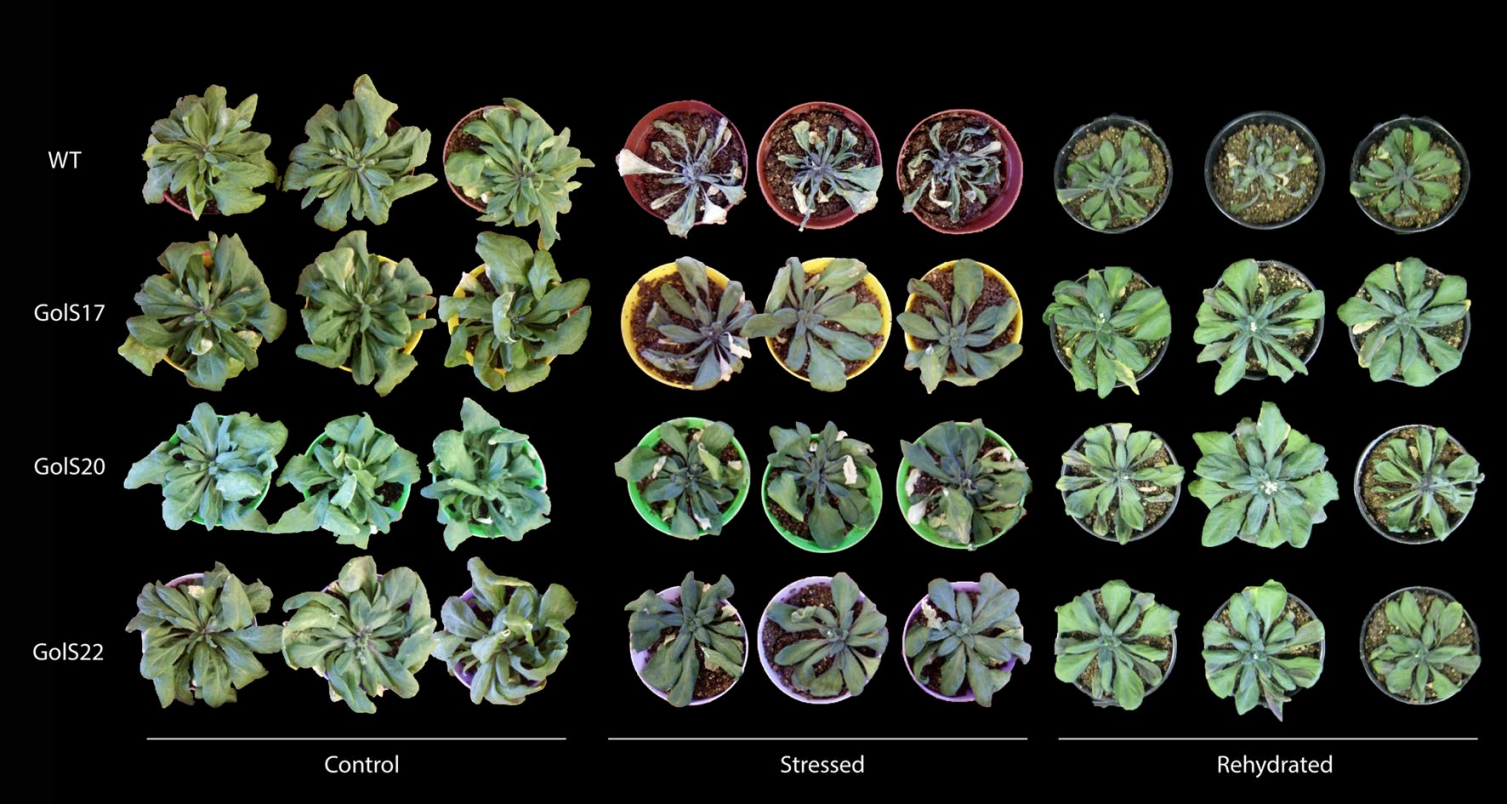
Phenotype of *A. thaliana* plants submitted to three treatments. Wild-type (WT) plants and three *AdGolS3* OE lines (GolS17, GolS20 and GolS22) submitted to three treatments: Control group maintained under irrigation (70% FC); Stressed group where irrigation was suspended for 20 days; and Rehydrated group where irrigation was suspended for 20 days followed by irrigation for 24 h. Each plant is a distinct individual and is representative of each treatment group.

Over the 20 days of the dry-down assay, the morphology of the aerial part of the *AdGolS3* OE lines remained similar to that of WT plants under normal irrigation conditions (CTR group) (Fig. 3). In the STR and REH groups, WT plants displayed severe morphological damage (leaf wilt) after 20 days without irrigation, whereas OE lines exhibited fewer symptoms of water deficiency (Fig. 3). In the REH group, one day after rehydration, transgenic plants recovered faster than WT, indicating that the *AdGolS3* overexpression increased the ability of plants to recover their normal phenotype, as that observed in CTR group.

**Figure 4:**
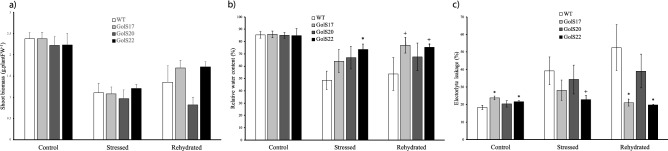
Performance of *A. thaliana* plants under the dry-down treatment. Wild-type (WT) plants and three *AdGolS3* OE lines (GolS17, GolS20 and GolS22) submitted to a dry-down treatment for 20 days (Stressed group), followed by 24 h of rehydration (Rehydrated group) and the corresponding irrigated (70% FC) control (Control group). (**a**) Shoot biomass (grams of fresh weight per plant). (**b**) Percentage of leaf relative water content (RWC). (**c**) Percentage of leaf electrolyte leakage (EL). Bars represent value means and standard errors. * and + indicate statistically significant differences compared to control WT plants for each OE line (T-test, n = 7–10, p < 0.05 and p < 0.1 respectively).

Plants submitted to dehydration for 20 days (STR and REH groups) had less shoot biomass when compared to the CTR group, with no significant differences between WT and OE lines (Fig. 4a). The analysis of the leaf relative water content (RWC) in plants maintained under CTR conditions showed values of 85–86%, typical of turgid leaves, with no differences between WT and OE lines (Fig. 4b). An overall reduction in RWC was observed when plants were submitted to STR conditions when compared to CTR. Under dehydration imposition, the three OE lines displayed RWC values higher than WT plants, with the GolS22 OE line showing a significant difference of 25%. Following 24 h of rehydration, both GolS17 and GolS22 OE lines reached higher RWC values than WT plants, comparable to those found for plants under CTR conditions, indicating their ability to rapidly recover to a high leaf water status in response to water availability (Fig. 4b). Accordingly, the opposite behavior was observed for leaf electrolyte leakage (EL) measurements at the end of the dry-down assay, with a significantly lower leakage in the GolS22 line under stressed conditions and in the GolS17 and GolS22 lines following rehydration, as compared to the WT plants (Fig. 4c), representing good evidence of cell membrane stability under stress in these OE lines. Interestingly, GolS17 and GolS22 lines showed significant differences in EL values compared to WT plants, even when plants were maintained under CTR conditions (Fig. 4c). RWC and EL measurements have been widely used to reflect, respectively, water loss control and membrane stability in plants submitted to water-limited conditions, and therefore an improvement in drought tolerance. These results could thus indicate that *AdGolS3* overexpression increases plant tolerance to water deficit stress.

#### Soluble sugar content and metabolic profiling

Soluble sugar (glucose, sucrose and raffinose) abundance in leaves of WT and OE lines (GolS17, GolS20 and GolS22) under CTR, STR and REH conditions were also analyzed. Despite the highly variable absolute concentration of these sugars, previously determined for the 13 OE lines under CTR condition only (Fig. S7), the relative percentage abundance was generally stable in the GolS17, GolS20 and GolS22 lines, regardless of the treatment applied (Fig. S8). The abundance of raffinose relative to glucose and sucrose was indeed higher in the GolS17 and GolS22 OE lines compared to WT plants under CTR conditions. These results corroborated the previous analysis of the absolute raffinose concentration (Figs. S7 and S8). Also, the relative abundance of raffinose did not differ between GolS20 and WT plants under CTR conditions, but it significantly increased from 2.5 to 12.9% when GolS20 plants was submitted to STR conditions (Fig. S8). Conversely, the relative abundance of raffinose in the GolS17 OE line significantly decreased from 7.6% of total sugars under CTR conditions to 3.9% when plants were rehydrated. Interestingly, the ratio between glucose and sucrose relative abundance was almost stable (around 1:1), and apparently independent of the treatment (CRT, STR or REH). In contrast, the ratio between raffinose and these sugars was more variable (Fig. S8).

To further characterize the metabolic response of the *AdGolS3* OE lines under STR and REH conditions, we carried out GC–MS based metabolite profiling of leaf tissues (Table 2 and Fig. S9). Overall, few differences between the WT and OE lines were detected in the metabolic profile regardless of the treatment. Under CTR conditions, GolS17 contained less sucrose whilst GolS22 contained more raffinose and galactinol than WT, mirroring the results obtained by HPAE (Table 2; Figs. S7, S8 and S9a). When expressed relative to all detected metabolites abundances of both raffinose and galactinol were also greater in GolS17 than in WT (Table 2 and Fig. S9b), again reflecting a shift in metabolite profile towards raffinose accumulation. As well as these alterations in sugars, citrate concentrations were also higher in GolS20 and threonate concentrations lower in GolS22 under CTR conditions. Under STR condition, the only difference was an increased concentration of putrescine in the GolS22 line compared to WT. Under the REH, when expressed relative to all detected metabolites, raffinose and galactinol in GolS22 remained greater than in WT (Table 2 and Fig. S9).

**Table 2:** Metabolic profiling in *AdGolS3*-OE lines.

		Control (CTR)								Stressed (STR)								Rehydrated (REH)							
		WT		GolS17		GolS20		GolS22		WT		GolS17		GolS20		GolS22		WT		GolS17		GolS20		GolS22	
		AVG	SD	AVG	SD	AVG	SD	AVG	SD	AVG	SD	AVG	SD	AVG	SD	AVG	SD	AVG	SD	AVG	SD	AVG	SD	AVG	SD
Amino acids	Alanine	1.0	0.4	1.1	0.4	1.1	0.4	1.0	0.4	4.4	2.2	4.4	1.6	3.3	3.0	3.5	2.3	5.5	5.0	2.2	1.4	2.2	1.6	1.2	0.7
	β-Alanine	1.0	0.3	1.0	0.1	1.1	0.4	1.0	0.3	1.8	0.4	2.0	0.3	1.5	0.4	1.6	0.4	1.6	0.5	1.3	0.4	1.2	0.7	1.0	0.4
	Asparagine	1.0	1.3	3.7	4.4	4.5	4.1	0.5	0.6	8.6	13.2	4.3	2.1	2.1	2.0	9.3	8.9	1.6	0.8	2.3	2.5	0.7	1.1	1.8	1.3
	Aspartate	1.0	0.8	2.2	2.0	4.3	3.1	0.6	0.5	5.2	2.0	8.2	4.2	2.4	1.9	7.0	5.4	3.5	2.1	3.6	3.3	1.9	2.7	2.1	1.7
	Glutamate	1.0	0.8	1.4	1.2	2.4	1.7	0.6	0.2	2.1	2.0	2.1	0.3	1.6	0.7	3.2	2.5	0.9	0.6	1.5	0.8	0.6	0.4	1.4	0.7
	Glutamine	1.0	1.2	2.6	2.7	3.5	3.5	0.2	0.2	2.7	3.3	3.7	2.7	2.1	2.0	8.5	9.9	0.8	0.3	1.5	1.4	0.7	1.0	1.4	1.7
	Glycine	1.0	0.3	1.2	0.5	0.9	0.2	0.7	0.3	8.1	11.5	7.1	8.4	2.7	3.1	1.6	0.8	16.6	18.5	10.5	13.5	7.1	5.7	3.1	2.4
	Leucine	1.0	0.9	1.4	1.1	2.0	1.5	1.3	0.8	9.0	5.9	8.2	2.1	5.1	2.5	7.1	2.4	4.1	2.7	2.2	1.6	2.7	2.1	1.8	2.0
	Proline	1.0	1.8	0.6	0.5	1.2	1.4	0.4	0.3	349.2	319.9	288.6	174.3	76.0	124.1	128.0	176.1	306.8	319.4	143.9	145.4	172.3	197.6	87.4	159.4
	Serine	1.0	0.7	4.4	6.8	6.4	6.1	2.5	2.5	20.3	22.0	20.0	8.3	6.0	4.2	19.3	15.1	10.6	7.9	9.1	9.0	8.7	9.2	6.3	7.6
	Threonine	1.0	0.6	2.2	2.7	3.2	3.0	2.4	1.6	11.4	10.7	10.3	3.1	3.9	2.0	8.4	5.1	6.6	3.6	6.5	5.1	5.1	5.3	5.1	5.8
	Valine	1.0	0.2	1.1	0.1	1.3	0.5	1.2	0.5	8.6	5.8	6.5	1.7	3.6	2.0	5.0	3.0	3.6	2.2	2.0	1.1	2.3	1.7	1.5	1.2
Organic acids	Citrate	1.0	0.6	1.4	1.1	**3.0**	**1.3**	1.5	0.9	2.4	0.8	2.9	0.7	2.5	1.6	2.6	1.1	2.8	1.3	3.2	4.2	1.5	0.6	1.9	0.5
	Fumarate	1.0	0.9	0.5	0.5	0.9	0.5	0.7	0.5	1.3	0.6	1.5	0.7	1.7	1.0	1.1	0.8	1.4	1.1	1.3	0.6	1.0	0.4	1.6	0.6
	Gluconate	1.0	0.8	1.8	1.1	1.4	1.7	1.8	1.5	26.7	22.4	21.5	9.1	9.9	7.5	10.7	5.9	117.2	163.3	45.1	61.8	57.7	67.6	8.4	4.2
	Malate	1.0	1.2	0.8	0.5	1.1	0.7	0.6	0.4	27.0	26.0	23.3	17.9	7.5	9.7	12.5	14.8	15.8	14.9	7.3	6.4	6.4	7.4	3.6	3.9
	Succinate	1.0	0.7	0.9	0.6	1.2	0.3	0.7	0.4	1.0	0.6	1.4	0.7	1.7	1.0	1.2	0.8	1.4	0.6	2.0	1.0	1.2	0.4	1.8	0.8
	Threonate	1.0	0.1	1.6	1.2	1.8	1.0	**0.8**	**0.1**	2.7	1.1	2.6	1.3	2.0	1.3	1.9	1.0	3.9	2.6	2.3	1.3	2.7	2.1	1.8	1.0
Sugars	Fructose 1	1.0	0.4	0.9	0.6	0.7	0.4	0.6	0.2	1.6	0.3	1.9	0.5	1.3	0.7	1.7	0.8	2.9	3.0	1.3	1.4	1.8	1.5	0.5	0.3
	Fructose 2	1.0	0.4	1.0	0.6	0.7	0.4	0.6	0.2	1.6	0.3	1.9	0.5	1.3	0.6	1.7	0.7	3.0	3.0	1.3	1.3	1.9	1.5	0.5	0.2
	Galactinol	1.0	1.0	1.6	0.8	0.6	0.3	**6.7**	**2.7**	3.7	1.9	9.2	6.4	5.5	1.8	11.6	7.4	2.8	2.7	2.2	0.7	2.6	2.3	**9.5**	**4.9**
	Glucose 1	1.0	0.6	0.6	0.3	0.5	0.3	0.5	0.2	3.3	1.8	3.7	1.5	2.0	1.4	2.0	1.7	2.6	2.3	1.5	0.8	2.4	1.9	1.7	1.2
	Glucose 2	1.0	0.6	0.6	0.3	0.5	0.3	0.5	0.1	3.8	2.4	4.4	2.3	2.2	1.8	2.1	1.8	2.7	2.6	1.6	0.9	2.5	2.0	1.8	1.4
	myo-Inositol	1.0	0.2	0.9	0.1	0.9	0.2	0.8	0.3	2.5	0.9	2.2	0.4	1.7	0.5	1.7	0.6	2.0	0.5	1.6	0.6	1.9	0.6	1.3	0.4
	Raffinose	1.0	0.7	1.6	0.8	0.7	0.3	**5.3**	**2.0**	4.3	2.3	6.7	2.2	5.2	1.6	9.0	5.0	2.1	1.6	1.3	0.6	1.8	1.6	3.9	1.5
	Sucrose	1.0	0.2	**0.6**	**0.2**	0.7	0.3	0.7	0.3	24.4	21.5	18.3	15.2	8.0	13.6	9.1	11.8	15.5	17.3	5.5	7.7	10.0	12.7	0.9	0.4
	Glycerol	1.0	0.8	0.8	0.8	2.6	1.9	1.9	0.9	7.7	4.4	6.8	2.5	3.8	2.0	4.4	3.6	12.1	12.1	5.2	4.4	8.2	6.1	3.5	1.3
	Putrescine	1.0	0.5	1.2	0.6	0.7	0.2	0.7	0.3	0.6	0.2	0.8	0.3	0.6	0.1	**0.9**	**0.2**	0.5	0.2	0.7	0.3	0.7	0.2	0.8	0.2

Relative abundance of metabolites extracted from leaves of wild-type (WT) untransformed plants and three OE lines (GolS17, GolS20 and GolS22) under control (CTR), stressed (STR) and rehydrated (REH) conditions. Values indicate the abundance of each metabolite relative to that detected in WT plants [average (AVG) and standard deviation (SD) of four to five individuals]. Values in bold are significantly different from WT plants for a given treatment, whilst underlined values are significantly different from the control treatment for a given genotype (WT or each OE line) (t-test, p < 0.05). Two chromatographic peaks were detected for fructose and glucose.

As anticipated, greater differences were observed between stressed and non-stressed control plants of the same genotype (WT and each OE line) (Table 2 and Fig. S9a). In stressed WT plants, for example, 13 out 26 metabolites showed increased concentrations, including alanine, beta-alanine, aspartate, citrate, fructose, galactinol, glucose, glycerol, myo-inositol, leucine, raffinose, threonate, and valine (Table 2 and Fig. S9a). GolS17 metabolism was similarly affected, with a total of 17 different metabolites showing significant alterations under stressed conditions. Fewer changes, however, were detected in both GolS20 and GolS22 OE lines, as only three (galactinol, myo-inositol and raffinose), and six (beta-alanine, fructose, gluconate, myo-inositol, leucine and valine) metabolites were significantly perturbed by the drought imposition, respectively. Myo-inositol was the only metabolite with increased abundance under stress in all genotypes (Table 2). Whilst large increases in proline were detected in individual samples, the only significant increase was in stressed GolS17 plants relative to their non-stressed controls. Rehydration generally led to metabolite concentrations returning to the levels observed in control conditions. One notable exception was that of myo-inositol, which remained at elevated concentrations relative to control plants for WT, GolS17 and GolS20 (Table 2).

#### NaCl and PEG treatments

**Figure 5:**
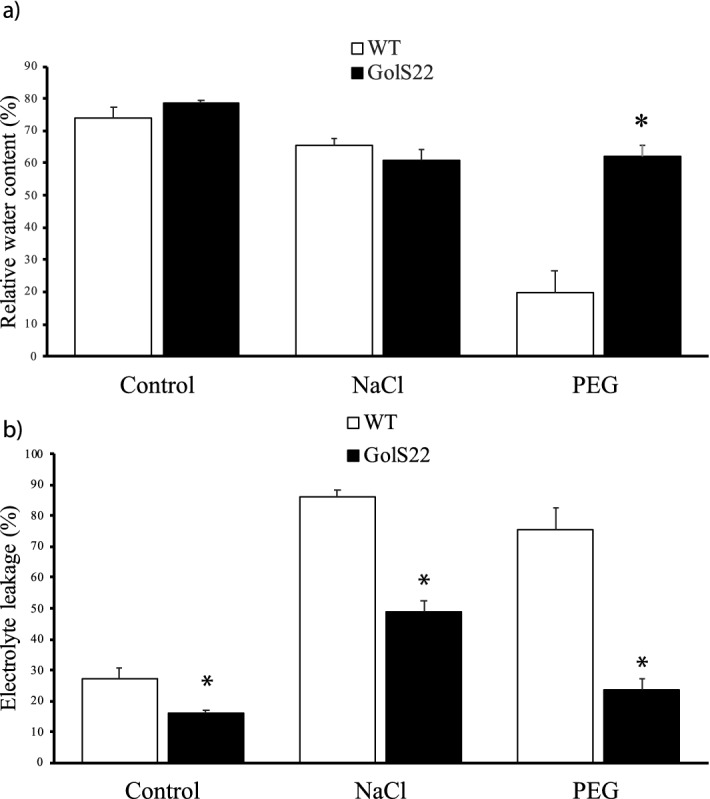
Performance of *A. thaliana* plants under the NaCl and PEG treatments. Wild-type (WT) plants and the *AdGolS3* OE line GolS22 submitted to salt stress (irrigated with 150 mM NaCl) and osmotic stress (irrigated with PEG 6,000 20%) treatments for 15 days and the corresponding irrigated (70% FC) Control. (**a**) Percentage of leaf relative water content (RWC). (**b**) Percentage of leaf electrolyte leakage (EL). Bars represent value means and standard errors. * indicate statistically significant differences compared to control WT plants for GolS22 OE line (T-test, n = 11 or 12, p < 0.05).

Additional analysis of GolS22 and WT plants grown under well-irrigated CTR, salt stress (irrigated with NaCl 150 mM) and osmotic stress (irrigated with PEG 20%) conditions was conducted after 15 days of treatment. The phenotype of the GolS22 transgenic plants was similar to that of WT under CTR conditions, with RWC values (78% and 74%, respectively) typical of turgid leaves (Fig. 5a). Similar RWC values for WT and GolS22 plants (65% and 61%, respectively) were maintained even when salt stress treatment was applied; however, RWC was significantly greater in GolS22 (62%) under osmotic stress (Fig. 5a). EL values in the GolS22 line were significantly lower than in WT plants under both stress conditions (NaCl and PEG) (Fig. 5b), as previously observed for drought and rehydration (Fig. 4c) treatments. Together, these results indicate that overexpression of *AdGolS3* led to better water retention and membrane stability not only during drought imposition and rehydration recovery but also under both salt and osmotic stresses.

#### qRT-PCR expression analysis

**Figure 6:**
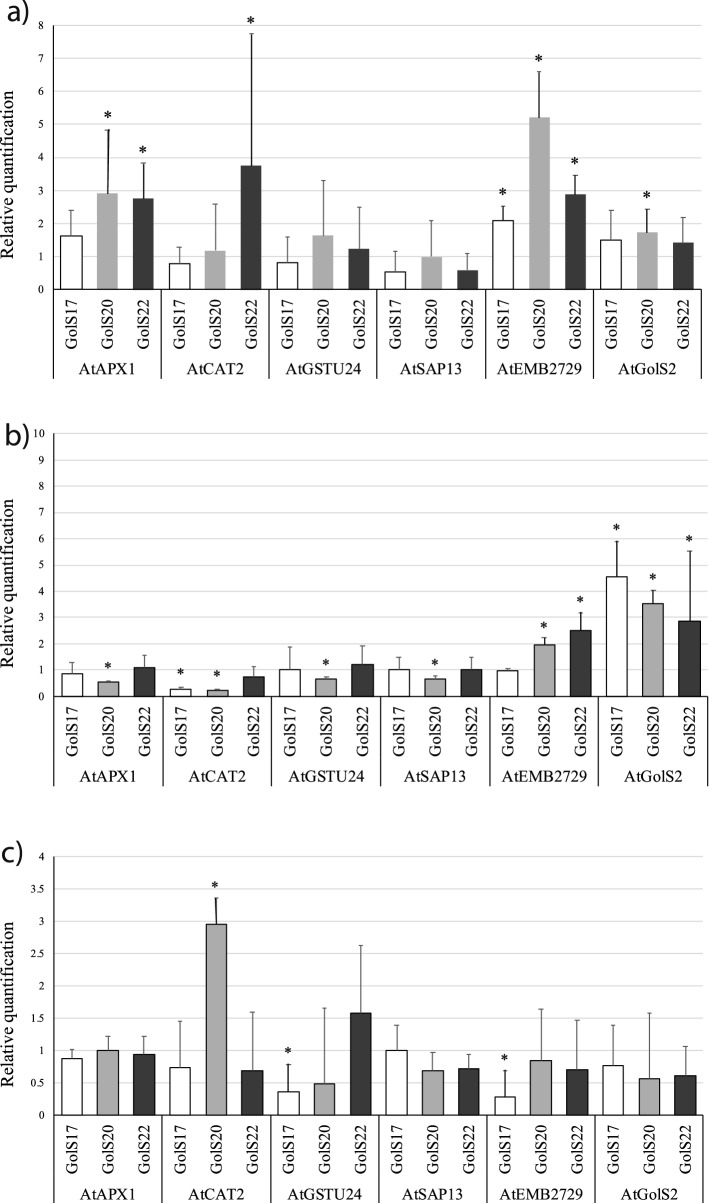
qRT-PCR expression analysis in *A. thaliana* plants. Relative quantification of mRNA levels of six *Arabidopsis* genes (*AtAPX1*, *AtCAT2*, *AtGSTU24*, *AtSAP13*; *AtEMB2729* and *AtGolS2*) in the three OE lines (GolS17; GolS20 and GolS22) relative to the wild-type (WT) plants, under (**a**) control, (**b**) stressed and (**c**) rehydrated conditions. Values are means ± SD of three biological replicates and the significant (p < 0.05) differences between WT and OE lines are marked with *.

Considering that *AdGolS3* overexpression enhanced drought tolerance in transgenic *Arabidopsis*, altering sugar and metabolic profiles, the expression levels of a subset of six genes was evaluated. This subset comprises the *AtGolS2* gene, which is the *AdGolS3* ortholog in *Arabidopsis*, and five other *Arabidopsis* genes coding for proteins that putatively interact with *AtGolS2* based on a predicted protein–protein interaction network (Fig. S10). These genes were also selected based on their known involvement in drought and osmotic stress responses. The expression analysis of the six genes was carried out by qRT-PCR in roots from three OE lines (GolS17, GolS20 and GolS22) and WT plants submitted to CTR, STR and REH conditions. As a whole, *AdGolS3* overexpression by itself was sufficient to induce the expression of five (*AtAPX1*, *AtCAT2*, *AtGSTU24, AtEMB2729* and *AtGolS2*) of the six *Arabidopsis* genes, given that their expression was higher in OE lines than in WT plants under CTR conditions. (Fig. 6a). Interestingly, under drought stress imposition, the expression behavior of four (*AtAPX1*, *AtCAT2*, *AtGSTU24* and *AtEMB2729*) of these genes changed drastically with a general pattern of downregulation, and an average decrease of almost threefold in transcript levels when compared to CTR conditions (Fig. 6b).

After subsequent plant rehydration, the expression levels of these four genes increased, almost reaching the basal levels observed under CTR conditions (Fig. 6c). Conversely, the endogenous *Arabidopsis GolS* gene (*AtGolS2*) was the only gene for which the expression increased (4.12-fold) under drought imposition as compared to CTR conditions, with significant upregulation in all OE lines (Fig. 6b). However, despite its remarkable upregulation under STR conditions, the expression of the *AtGolS2* gene rapidly dropped and returned to the basal CTR levels just 24 h after rehydration, as also observed for the other four genes (Fig. 6c). It is interesting that the expression of *AtSAP13* was negatively affected by *AdGolS3* overexpression, but not by dry-down or rehydration treatments, since its downregulation was maintained (average RQ of 0.81) in the three OE lines under all of the studied conditions (Fig. 6). These findings suggest that *AdGolS3* may play an important role in drought-associated pathways by modulating the transcriptional dynamics of downstream genes.

## Discussion

### The majority of RFO metabolism genes have undergone dispersed duplications in wild *Arachis*

RFOs are part of the molecular network activated by plants in response to a range of environmental stresses and currently emerge as key components in stress tolerance, acting as osmoprotectants, antioxidants and signaling molecules^2,4^. Due to their importance, the principal enzymes involved in the first steps of RFO biosynthesis (GolS, RS and STS) have been thoroughly studied at the genome-wide scale in many plant species^8–12,15^. However, little attention has been given to the enzymes involved in RFO catabolism, such as AGAL and BFLUCT. These enzymes are equally important for the accumulation of RFOs in plants, but only few reports provide a comprehensive analysis of genes involved in both RFO biosynthesis and catabolism^5,8,15^.

The availability of *A. duranensis* and *A. ipaënsis* genomes^25^*,* the progenitors of peanut (*A. hypogaea*), enabled the genome-wide search for gene families and the assessment of their evolutionary history in these wild species. Here, a comprehensive analysis of five gene families (GolS, RS, STS, AGAL and BFLUCT) was conducted, which lead to the identification of 28 genes related to both RFO biosynthesis and catabolism in *A. duranensis* and 31 in *A. ipaënsis*. This is in accordance with the 35 RFO-related genes described for *Z. mays*^8^ and the 58 RFO-related loci identified in *G. max*^15^.

The phylogenetic analysis of putative RFO-related proteins from Fabaceae, including these five enzyme families identified in wild *Arachis*, showed a clear subdivision within each family, supported by high bootstrap values. This clustering, based on phylogenetic proximity, was related to the intron/exon organization of the corresponding *Arachis* genes, as previously observed for other plant species^8–10^. Similarly to the conservation of gene structure, amino acid sequences were highly conserved too, for example, the hydrophobic pentapeptide (APSAA), characteristic of the GolS family^42^, was found for all the proteins from *A. duranensis* and *A. ipaënsis,* as well as other conserved motifs in the different enzyme families*.*

The highly duplicated state of the genes involved in raffinose metabolism is consistent with previous descriptions for other gene families in wild *Arachis*, such as expansin and NBS-LRR families^23,40^, however, this was not observed for the dehydrin family^22^. The majority of the wild *Arachis* RFO metabolism genes have undergone dispersed duplications. Conversely, the genes from the GolS family have undergone duplication mostly by WGD and can be observed in gene blocks in two different chromosomes. Other studies also found that segmental duplications were the driving force for the expansion of GolS genes in *S. indicum* and Rosaceae genomes, being associated with a possible subfunctionalization^10,12^.

### The expression profiling of wild *Arachis* genes differed according to the water-deficit treatment

Wild and cultivated *Arachis* genotypes exhibit contrasting transpiration behaviors under water-limited conditions, with variable levels of water stress tolerance among the wild species^43^. Accordingly, the accessions K7988 of *A. duranensis* and V10309 of *A. stenosperma* have been selected as the drought-tolerant and the drought-sensitive genotypes, respectively, in our functional genomics studies^18,20,21,23,24^. Here, the comprehensive expression analysis conducted in these genotypes revealed that most of the 28 *A. duranensis* genes involved in RFO metabolism, and their orthologs in *A. stenosperma*, are responsive to drought imposition.

Overall, the expression profiling of these genes indicated that, in wild *Arachis*, the molecular responses necessary to trigger RFO biosynthesis, accumulation, and eventually catabolism, differed according to the severity of the water loss process, as demonstrated for resurrection plants^44^. As GolS is the first committed enzyme and the key regulator of the RFO pathway, its general induction in *Arachis* roots submitted to dehydration could be a rapid transcriptional response to the severe process of water loss during air-drying. However, over the 150 min time frame of the experiment, increased levels of GolS transcripts seem to be insufficient to allow the observation of the expected increase in the expression of the downstream genes involved in the subsequent steps of RFO biosynthesis (*RS* and *STS*). Similar comprehensive expression profiles of *GolS, RS* and *STS* genes were observed in response to water-deficit treatments in *S. indicum*, *M. esculenta* and *G. max*^9,10,15^. Also, genes involved in RFO catabolism (*AGAL* and *BFLUCT*) were downregulated in response to this severe water-limited condition. It suggested the role of some members of these two multigene families in the transcriptional regulatory networks of drought tolerance in *Arachis*. Conversely, the moderate drought process imposed by four days of soil drying did not appear to affect the expression of *GolS* genes in *Arachis*. Under this moderate stress condition, the subsequent steps of the RFO metabolic pathway were also not yet induced, and accordingly, the transcript levels of genes coding for the enzymes that participate in RFO biosynthesis (*RS* and *STS*) and catabolism (*AGAL* and *BFLUCT*) were kept rather constant or slightly repressed.

### *AdGolS3* showed opposite expression behavior between drought-tolerant and drought-sensitive wild *Arachis*

The overexpression of *GolS* genes leads to enhanced tolerance to abiotic stresses (drought, salt, heavy metal, cold and heat), by increasing galactinol and RFO contents in transgenic dicots and monocot species^41,45–52^. These transgenes were isolated from a number of plant species being the *AtGolS2* gene from *Arabidopsis*, the most commonly used. An *AtGolS2* ortholog has been isolated from *Thellungiella salsuginea*, and its overexpression also resulted in improved tolerance to abiotic stress in transgenic plants^53^. Besides being the ortholog of *AtGolS2* in *A. duranensis*, the *AdGolS3* gene was selected for further functional analysis since it exhibited differential expression behavior between the drought-tolerant *A. duranensis* and the drought-sensitive *A. stenosperma,* in both severe and moderate water-limited conditions. Moreover, our previous qRT-PCR expression analysis^21^ showed that *AdGolS3* had a higher magnitude of expression in the tolerant *A. duranensis* throughout the dehydration treatment. Together, these findings indicate *AdGolS3* as a putative regulator gene of the RFO pathway in the *A. duranensis* drought tolerance mechanisms that could be involved in the early and differential responses to severe and moderate processes of water loss.

### Overexpression of *AdGolS3* in *Arabidopsis* increased water retention, maintained membrane integrity and altered metabolic profile

Under water deficit and PEG-mediated osmotic stress, and following rehydration, *Arabidopsis AdGolS3* OE lines exhibited higher RWC values than WT plants, indicating less water loss. The capacity of transgenic plants to maintain high leaf water status under abiotic stresses, as expressed in higher RWC, was also observed in some crop species overexpressing *GolS* genes, such as rice, poplar and chickpea^46,49,54^. This greater water retention in transgenic plants due to *GolS* overexpression may reflect a better uptake of soil water by roots and/or lower transpiration. It could increase their efficiency in the control of stomatal opening under water deficit conditions, as previously shown in transgenic *Brachypodium distachyon* plants^48^. We also observed a reduced EL when OE lines were submitted to drought, salinity and osmotic stresses, and following rehydration, indicating that *AdGolS3* overexpression attenuated the damage of cell membranes. The electrolyte leakage results from loss of cell membrane integrity caused by the generation of ROS in plants submitted to stress conditions and is commonly used to estimate the degree of the membrane injury. Our findings suggested that the overexpression of *AdGolS3* led to a common mechanism to respond to, at least, three different types of abiotic stresses (drought, salinity and osmotic), which involves better water retention and less damage to the plasma membrane, as previously suggested by^48,50,53^. The maintenance of plant water relations and cell membrane integrity have been considered important factors contributing to abiotic stress tolerance.

In addition, overexpression of *AdGolS3* resulted in *Arabidopsis* plants with increased concentrations of few metabolites, including galactinol, product of GolS enzyme activity, and a direct precursor of raffinose. Accordingly, leaf raffinose concentrations were also higher in OE lines than in WT plants as well as raffinose representing a greater proportion of leaf sugar. As expected, when drought stress was imposed the metabolic profile in WT plants changed drastically, with increased levels of most metabolites. Nevertheless, few metabolites were altered in GolS20 and GolS22 OE lines in response to drought stress, which exhibited an overall similar metabolic profile to non-stressed CTR conditions. This suggests that *AdGolS3* overexpression in these two lines may have led to a reduction in the metabolic perturbation caused by water deficit. Interestingly, qRT-PCR analysis showed that the expression of the endogenous *Arabidopsis AtGolS2* gene was also highly induced in response to drought stress in all OE lines compared to WT. It is likely to have contributed to increased galactinol accumulation with minor alterations in the metabolite profile of transgenic plants. The *AtGolS2* gene is known to be induced in *Arabidopsis* by drought and salinity stresses and is directly regulated by the heat shock transcription factors^41,55^.

Following rehydration, most metabolite concentrations in WT plants and OE lines returned roughly to levels found in non-stressed plants. However, myo-inositol concentrations remained elevated in WT plants and two of the OE lines, possibly due to its role as one of the principal metabolites, together with galactinol, of the classical RFO pathway^3^.

The ability of *AdGolS3* to improve the tolerance to three different types of abiotic stresses corroborates previous studies that signal GolS as a regulator of the synthesis of galactinol and raffinose, and indirectly other sugars, in stress-tolerant transgenic plants^41,46,48–53^. This may be related to the multiple putative functions of these oligosaccharides in plants. Raffinose may act to stabilize sensitive macromolecular structures and membranes under stress as well as act as an osmolyte^48^. Besides, such sugars represent also essential sources of energy, not only during germination but during recovery from a variety of abiotic stresses. Furthermore, galactinol and raffinose may also scavenge hydroxyl radicals, leading to oxidative stress defense^56–58^.

### Overexpression of *AdGolS3* in *Arabidopsis* modulated the expression of genes involved in plant protection against oxidative damage

Whilst ROS have important signaling roles in plant defense mechanisms, their increased production in response to stress can damage cellular components and is often accompanied by harmful effects on basic cellular processes^4, 59^. Plants have evolved detoxification mechanisms to control the excess accumulation of ROS that include antioxidant enzymes, such as catalase (CAT), ascorbate peroxidase (APX), and glutathione S-transferase (GST). CAT converts H_2_O_2_ into H_2_O and O_2_ and is induced by multiple abiotic stresses^60^. Likewise, APX is responsible for the removal of H_2_O_2_ and its activity increases in response to stress exposure whereas the knockout of its cytosolic isoforms reduced tolerance to a variety of abiotic stresses^61^. GSTs, such as that encoded by *AtGSTU24*, have both catalytic and non-catalytic activities that allow them to act in a variety of plant defense strategies, such as antioxidant regulation, adaptation and tolerance to abiotic constraints and pathogen resistance^62,63^.

Given that the *AdGolS3* OE lines exhibited increased stress tolerance, we analyzed the expression of genes coding for these three antioxidant enzymes in *Arabidopsis*. The expression behavior of *AtCAT2; AtGSTU24* and *AtAPX1* was similar, with increased transcript levels as a result of *AdGolS3* overexpression, which dropped to a pattern of downregulation when plants were submitted to STR and REH conditions.

The overexpression of *AdGolS3* therefore coincided with accumulation of transcripts of at least the three antioxidant enzyme-coding genes that could potentially increase plant capacity to detoxify ROS, as indicated by their expression profile under CTR conditions, though the mechanistic link between these observations is unclear. Moreover, and consistent with our analysis of electrolyte leakage, lower antioxidant enzyme transcript levels under drought stress suggests that the *AdGolS3* overexpression plants suffer from less oxidative than WT plants. This could be due to the action of RFOs as non-enzymatic antioxidants^56,64^, or due to their action in maintaining plant water content and protecting against the negative effects of desiccation. Additional experiments, including measurement of antioxidant enzyme activities and ROS quantification, will be required to determine how RFOs act in the context of *AdGolS3* overexpression.

### The potential roles of *AtSAP13* and *AtEMB2729,* genes with altered expression in *Arabidopsis* OE lines

Following identification of proteins that putatively interact with *AtGolS2*, we carried out expression analysis of a member of the “Stress-Associated Protein” family (*AtSAP13*) and a member of the alpha-amylase protein family (*AtEMB2729*). These genes displayed opposite expression behaviors, with *AtSAP13* consistently being downregulated in OE lines regardless of the conditions studied (CTR, STR and REH), and *AtEMB2729* being induced under these conditions. Whilst the mechanism of action of SAP family, a class of zinc-finger proteins with A20/AN1 domains, remains unknown, they have been considered as novel regulators (positive or negative) of stress signaling mediated by abscisic acid (ABA) and some representatives of the SAP family displayed a negative role in stress tolerance, by increasing plant sensitivity to drought, cold and salinity^65,66^. Likewise, little is known about the contribution of the *AtEMB2729* gene (also designated as *branching enzyme 1*, *BE1*) to abiotic stress responses^67^. Besides playing a critical function in embryogenesis and maintenance of carbohydrate homeostasis, *BE1* genes may also act in the regulation of auxin and cytokinin metabolism^67,68^. The induction of *AtEMB2729* in *AdGolS3* OE lines was consistent with a potential role in regulating sugar metabolism under abiotic stress conditions. Elucidation of the function of these genes in stress tolerance and their functional relationship with *AtGolS2* and RFO metabolism will, however, require further investigation including phytohormone profiling.

### Improved tolerance to abiotic stresses and accumulation of antinutritional effects

In the present study, the overexpression of the *AdGolS3* gene increased the abundance of galactinol and raffinose in transgenic *Arabidopsis* plants and conferred tolerance to drought, salt and osmotic stresses. These results therefore corroborate previous reports demonstrating that the overexpression of *GolS* genes can successfully impart tolerance to multiple abiotic stresses in the models *Arabidopsis* and tobacco^5^ and also in crop species such as rice, soybean, tomato, and poplar^45,46,49,52^. It is worth noting, however, that the natural accumulation of RFOs in mature seeds to protect the embryo from desiccation is considered an antinutritional factor for many grain legumes^13,17^. Thus, the reduction of RFO content, or even their removal, to improve digestibility and nutritional quality of seeds, has been the focus of many legume breeding programs^16^. It includes the development of transgenic plants by the antisense/RNAi suppression of RFO-synthesizing genes or by the overexpression of RFO-degrading genes^69^. Therefore, the consistent benefits regarding abiotic stress tolerance of transgenic plants overexpressing *GolS* genes could come with the potential drawback: higher contents of RFOs in their mature seeds.

## Conclusions

In the present work, we studied five key enzymes (GolS; RS; STS; AGAL and BFLUCT) involved in the biosynthesis and catabolism of RFOs in legumes, with an emphasis on wild relatives of peanut, *A. duranensis* and *A. ipaënsis.* This represents the first genome-wide survey of genes associated with RFO metabolism in wild *Arachi*s, including those responsive to drought. Furthermore, the overexpression of *AdGolS3* gene, encoding a galactinol synthase isolated from *A. duranensis*, led to increased raffinose production and tolerance to drought, salt and osmotic stresses in transgenic *Arabidopsis*. In addition to alterations in metabolite profile, the overexpression of *AdGolS3* modulated the expression of antioxidant genes, suggesting a protective effect in preventing oxidative damage of plant cells. *AdGolS3* is, therefore, a promising candidate gene for introduction in transgenic crops to increase their tolerance to abiotic stresses, though potential impacts on seed nutritional quality must be considered, particularly in legume crop engineering.

## Supplementary information

Supplementary file1
