# Supplementary material for: Characterization of raffinose metabolism genes uncovers a wild Arachis galactinol synthase conferring tolerance to abiotic stresses
Source: Sci Rep. 2020 Sep 17;10:15258. doi: 10.1038/s41598-020-72191-4 (PMC7498584; doi:10.1038/s41598-020-72191-4)
Supplement: Supplementary file 1 — Supplementary file1 [file 41598_2020_72191_MOESM1_ESM.pdf]

# Characterization of raffinose metabolism genes uncovers a wild Arachis galactinol synthase conferring tolerance to abiotic stresses

Christina C. Vinson[1,2], Ana P. Z. Mota[1], Brenda N. Porto[1], Thais N. Oliveira[1], Iracyara Sampaio[1,2], Ana L. Lacerda[1] , Etienne G. J. Danchin[3], Patricia M. Guimaraes[1] , Thomas C. R. Williams[1], Ana C. M. Brasileiro[1*].

Affiliations:

[1] EMBRAPA Recursos Genéticos e Biotecnologia. Parque Estação Biológica, CP 02372. Final W5 Norte, Brasília, DF – Brazil;
[2] Departamento de Botânica, Universidade de Brasília, Campus Darcy Ribeiro, Brasília, DF – Brazil;
[3] INRA, Université Côte d'Azur, CNRS, ISA, Sophia-Antipolis, France.
* Corresponding author: Telephone number: +55.61.3448-4901; Fax number: +55.61.3340-3658; e-mail address: ana.brasileiro@embrapa.br.

**Supplementary material**

## 1. Supplementary Methods

### *1.1.Sequence alignment and phylogenetic analysis*

Multiple sequence alignment of all proteins identified in Fabaceae species as GolS, RS, STS, AGAL, and BFLUCT was performed using MAFFT[1] and trimmed with trimAL[2]. Phylogenetic trees were constructed using RAxML using the methodology and parameters previously described by[3]. The tree was visualized with the iTOL web interface version 4.1 (https://itol.embl.de/).

### 1.2. *Gene/protein structures and genomic distribution in Arachis* spp.

The exon/intron structure of Arachis genes were retrieved from PeanutBase (https:/peanutbase.org/) and submitted to the Gene Structure Display Server (GSDS) software version 2.0 (http://gsds.gao-lab.org/index.php) for graphical representation. The corresponding protein sequences were submitted to the MEME-suite (http://meme-suite.org) to identify the conserved motifs within each enzyme family. InterPro (http://www.ebi.ac.uk/interpro/search/sequence) and SignalP (http://www.cbs.dtu.dk/services/SignalP) software programs were used for protein domain and signal peptide prediction, respectively.

The physical location of GolS, RS, STS, AGAL and BFLUCT genes in the *A. duranensis* and *A. ipaënsis* chromosomes were obtained from the gene model annotations at PeanutBase (http://peanutbase.org). The software MCScanX[4] was used to analyze the syntenic relationships and duplication patterns between the two *Arachis* species using default parameters. The results were visualized in Circa software (http://omgenomics.com/).

**Table S1.** Primers used for qRT-PCR analysis.

| Primer name | Gene description | Gene ID | Primer sequence (5´ - 3´) Forward/Reverse | Reference |
|---|---|---|---|---|
| eGFP | Enhanced Green Fluorescent Protein | XM_013480425[a] | GAGCTGAAGGGCATCGACTT/ TTCTGCTTGTCGGCCATGAT | [5] |
| AdGolS3 | Galactinol Synthase | Aradu.ZK8VV[b] | GCAGTCCATGACAGCGTAGA/ ACCCAGTTTGCCATGGCTTA | [6] |
| AtGSTU24 | Glutathione S-Transferase | AT1G17170[c] | GTGAATGTTACGGCGAGAAGG/ TACTCCAACCCAAGTTTCTTCCTA | [7] |
| AtSAP13 | Stress-Associated Protein | AT3G57480[c] | TGGTCTAGTCTTTTCGCTTCTG/ CATCAACCGTAACTTTGCCAC | [8] |
| AtAPX1 | Ascorbate Peroxidase 1 | AT1G07890[c] | GCATGGACATCAAACCCTCT/ AGCAAACCCAAGCTCAGAAA | This study |
| AtCAT2 | Peroxisomal Catalase 2 | AT4G35090[c] | TCAAGGAAGAAGGAGCTTTCA/ CATCATGTGGATCTTCTGTGC | [9] |
| AtEMB2729 | Alpha Amylase Family Protein | NM_001161157.2[a] | TCAATCACGCCTTCACAGAG/ AACATCAGTCGGCCTACACC | This study |
| AtGolS2 | Galactinol Synthase 2 | AT1G56600[c] | ACTACGTGAAAGGGGTGGTG/ CAACTGTTTCCGGTGATCCT | This study |
| AtACT2 | Actin 2 | AT3G18780[c] | CTTGCACCAAGCAGCATGAA/ CCGATCCAGACACTGTACTTCCTT | [10] |
| AtEF-1α | Elongation Factor-1α | AT5G60390[c] | TGAGCACGCTCTTCTTGCTTTCA/ GGTGGTGGCATCCATCTTGTTACA | [10] |

[a] NCBI: National Center for Biotechnology Information (http://www.ncbi.nlm.nih.gov);

[b] PeanutBase: Bioinformatic Resources for Peanut (https://www.peanutbase.org/);

[c] TAIR: The Arabidopsis Information Resource (https://www.arabidopsis.org/).

**Table S2.** Distribution in Fabaceae species of proteins identified as putatively belonging to the five enzyme families involved in RFO metabolism (GolS; RS; STS; AGAL and BFLUCT).

| Fabaceae species | Code | GolS | RS | STS | AGAL | BFLUCT | TOTAL |
|---|---|---|---|---|---|---|---|
| *Arachis duranensis* | Ad | 5 | 7 | 1 | 4 | 11 | 28 |
| *Arachis ipaënsis* | Ai | 5 | 6 | 3 | 4 | 13 | 31 |
| *Cajanus cajan* | CAJCA | 2 | 6 | 3 | 2 | 6 | 19 |
| *Cicer arietinum* | CICAR | 4 | 7 | 1 | 4 | 11 | 27 |
| *Cyamopsis tetragonoloba* | CYATE | 0 | 0 | 0 | 1 | 0 | 1 |
| *Glycine max* | GLYMA | 6 | 9 | 0 | 8 | 20 | 43 |
| *Kummerowia stipulacea* | KUMST | 0 | 0 | 0 | 0 | 4 | 4 |
| *Lupinus angustifolius* | LUPAN | 4 | 7 | 1 | 6 | 10 | 28 |
| *Medicago sativa* | MEDSA | 1 | 0 | 0 | 0 | 0 | 1 |
| *Medicago truncatula* | MEDTR | 3 | 12 | 0 | 9 | 10 | 34 |
| *Phaseolus angularis* | PHAAN | 3 | 3 | 0 | 3 | 6 | 15 |
| *Phaseolus vulgaris* | PHAVU | 2 | 4 | 0 | 5 | 13 | 24 |
| *Pisum sativum* | PEA | 1 | 2 | 1 | 1 | 7 | 12 |
| *Trifolium pratense* | TRIPR | 0 | 3 | 1 | 4 | 8 | 16 |
| *Trifolium subterraneum* | TRISU | 3 | 5 | 1 | 4 | 11 | 24 |
| *Vicia faba* | VICFA | 0 | 0 | 0 | 0 | 2 | 2 |
| *Vigna radiata* | VIGRR | 1 | 4 | 2 | 1 | 6 | 14 |
| TOTAL | | 40 | 75 | 14 | 56 | 138 | 323 |

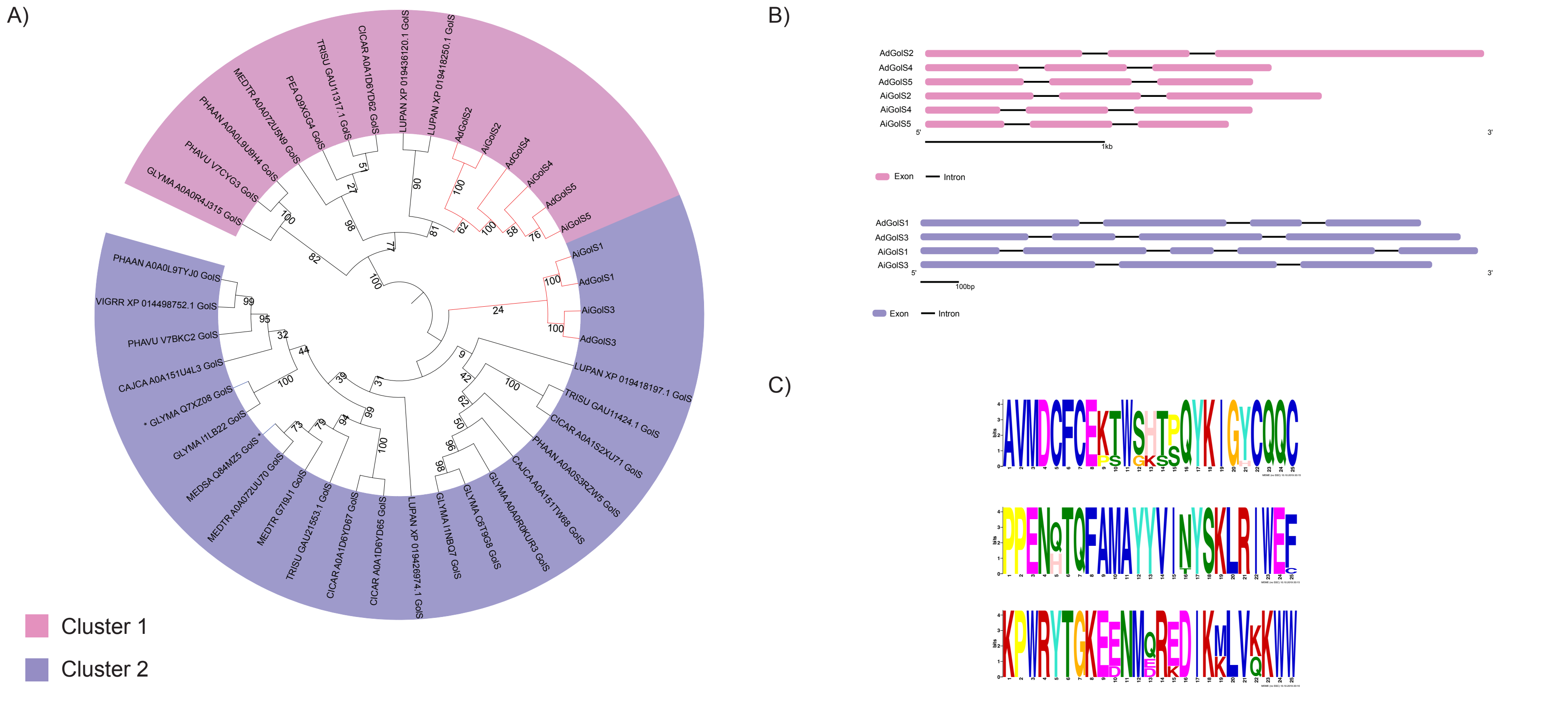


**Figure S1.** Phylogenetic analysis of the GolS proteins from the Fabaceae family and gene/protein organization of the 10 *Arachis GolS* genes. **A.** Phylogenetic analysis of the 40 putative GolS proteins in 13 Fabaceae species. Blue lines and asterisk show the reference protein from CAZy and the red lines the genes of *Arachis* spp. The tree was visualized using iTol (https://itol.embl.de). **B.** Exon/intron gene structure (visualized in GSDS software in http://gsds.gao-lab.org) and **C.** Protein motif organization of the 10 *GolS* genes in *Arachis duranensis* and *A. ipaënsis*. The motif search was performed by MEME-suite (http://meme-suite.org/tools/meme). The scale length is represented in base pairs (bp) and the introns are rescaled to have the same length.

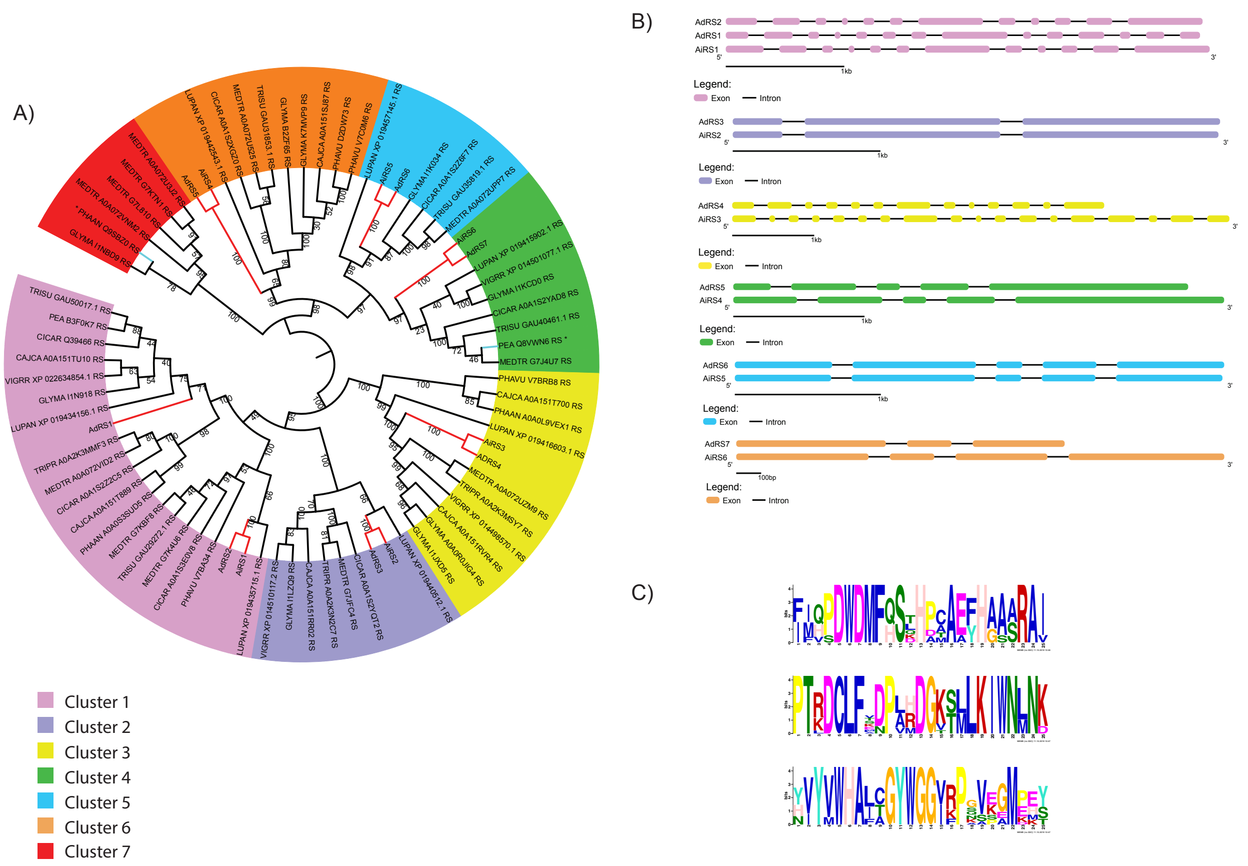


**Figure S2.** Phylogenetic analysis of the RS proteins from the Fabaceae family and gene/protein organization of the 13 *Arachis* RS genes. **A.** Phylogenetic analysis of the 75 putative RS proteins in 13 Fabaceae species. The tree was visualized using iTol (https://itol.embl.de). Blue lines and asterisk show the reference protein from CAZy and the red lines the genes of *Arachis* spp. **B.** Exon/intron gene structure (visualized in GSDS software in http://gsds.gao-lab.org) and **C.** Protein motif organization of the 13 RS genes in *Arachis duranensis* and *A. ipaënsis.* The motif search was performed by MEME-suite (http://meme-suite.org/tools/meme). The scale length is represented in base pairs (bp) and the introns are rescaled to have the same length.

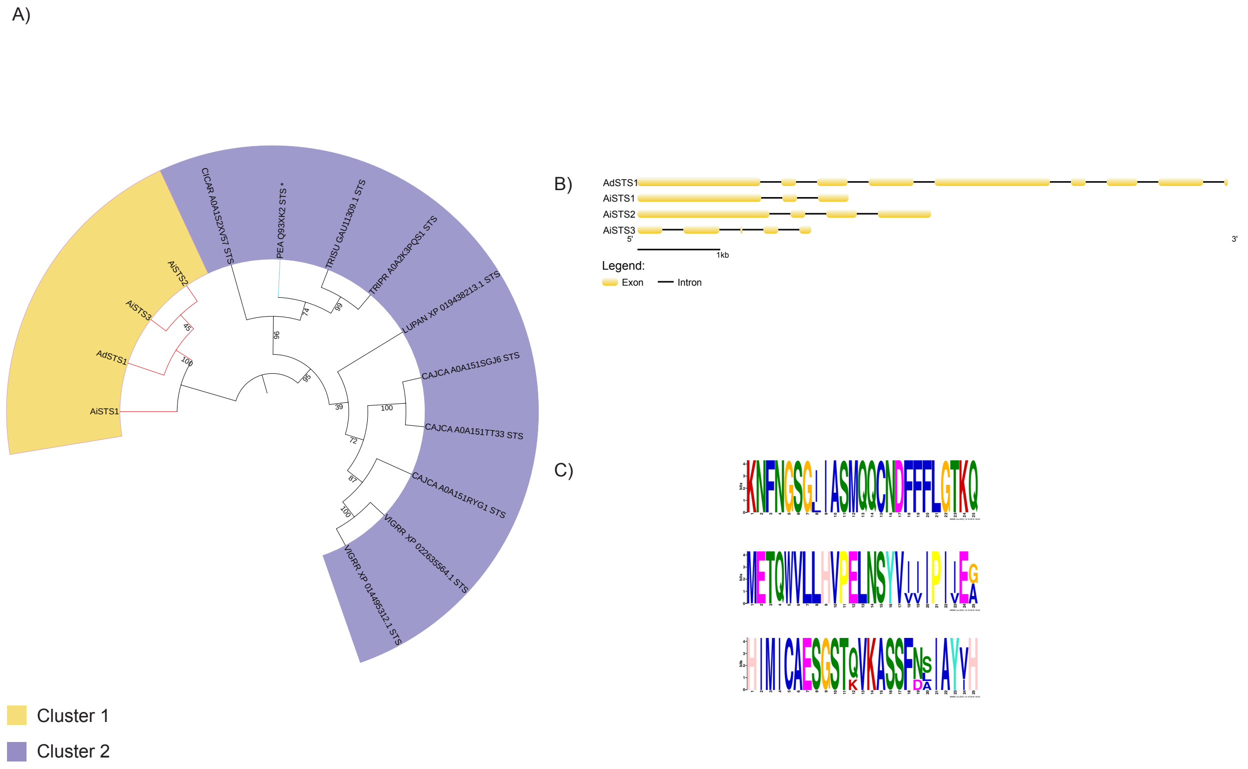


**Figure S3.** Phylogenetic analysis of the STS proteins from the Fabaceae family and gene/protein organization of the four *Arachis* STS genes. **A.** Phylogenetic analysis of the 14 putative STS proteins in eight Fabaceae species. The tree was visualized using iTol (https://itol.embl.de). Blue lines and asterisk show the reference protein from CAZy and the red lines the genes of *Arachis* spp. **B.** Exon/intron gene structure (visualized in GSDS software in http://gsds.gao-lab.org) and **C.** Protein motif organization of the four STS genes in *Arachis duranensis* and *A. ipaënsis*. The motif search was performed by MEME-suite (http://meme-suite.org/tools/meme). The scale length is represented in base pairs (bp) and the introns are rescaled to have the same length.

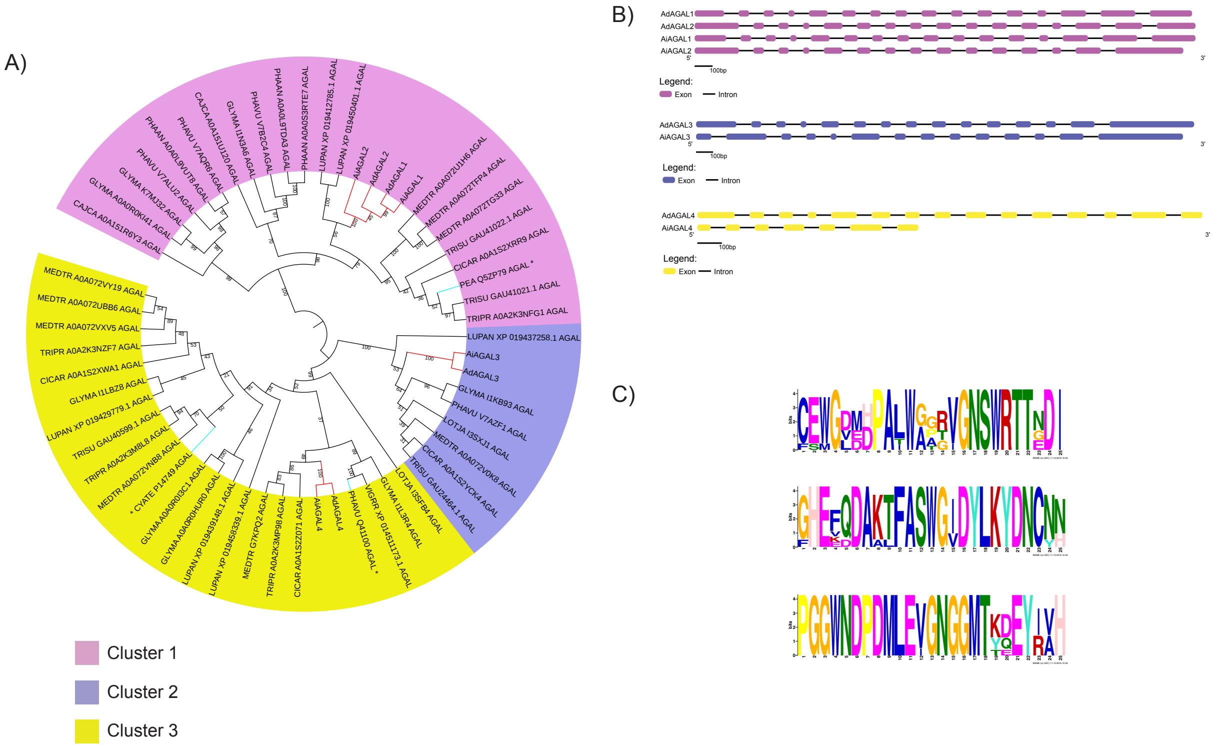


**Figure S4.** Phylogenetic analysis of the AGAL proteins from the Fabaceae family and gene/protein organization of the eight *Arachis* AGAL genes. **A.** Phylogenetic analysis of the 56 putative AGAL proteins in 14 Fabaceae species. The tree was visualized using iTol (https://itol.embl.de). Blue lines and asterisk show the reference protein from CAZy and the red lines the genes of *Arachis* spp. **B.** Exon/intron gene structure (visualized in GSDS software in http://gsds.gao-lab.org) and **C.** Protein motif organization of the eight AGAL genes in *Arachis duranensis* and *A. ipaënsis.* The motif search was performed by MEME-suite (http://meme-suite.org/tools/meme). The scale length is represented in base pairs (bp) and the introns are rescaled to have the same length.

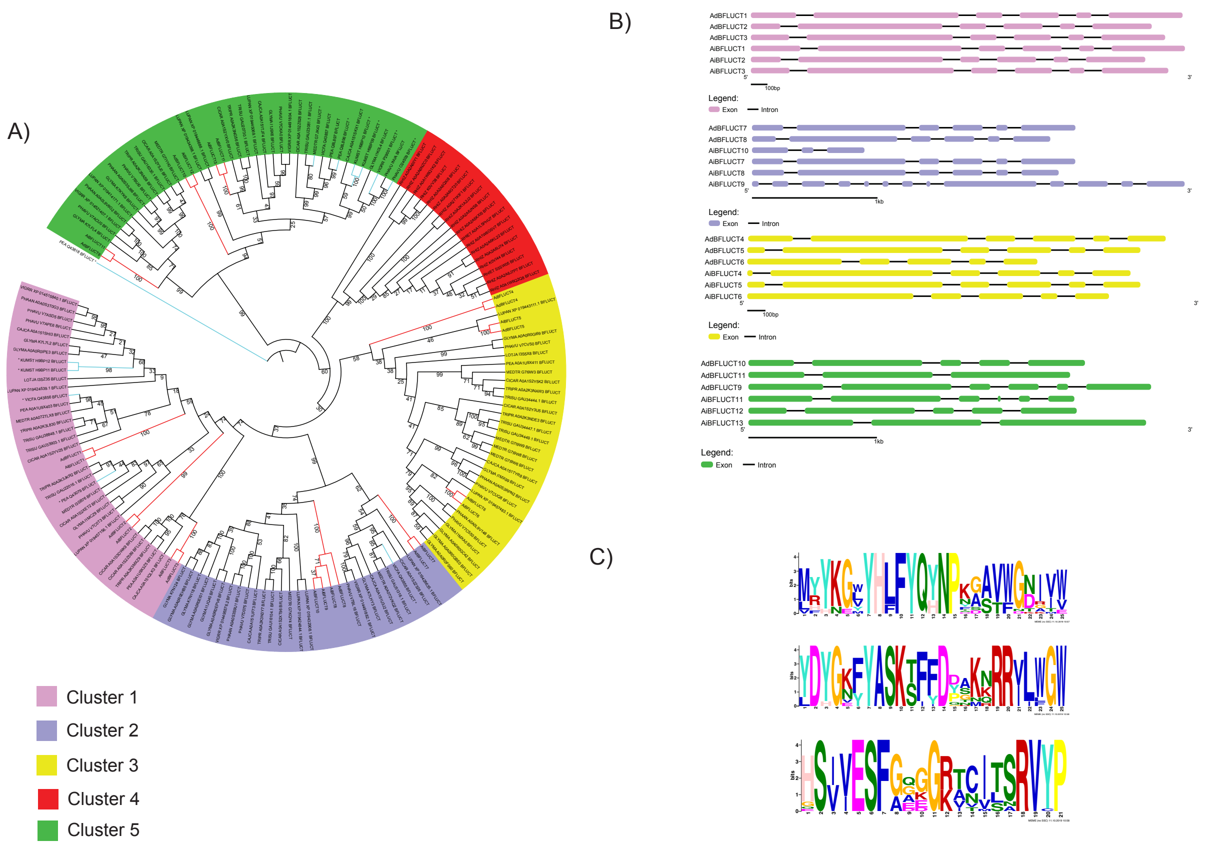


**Figure S5.** Phylogenetic analysis of the BFLUCT proteins from the Fabaceae family and gene/protein organization of the 24 *Arachis* BFLUCT genes. **A.** Phylogenetic analysis of the 138 putative BFLUCT proteins in 15 Fabaceae species. The tree was visualized using iTol (https://itol.embl.de). Blue lines and asterisk show the reference protein from CAZy and the red lines the genes of *Arachis* spp. **B.** Exon/intron gene structure (visualized in GSDS software in http://gsds.gao-lab.org) and **C.** Protein motif organization of the 24 BFLUCT genes in *Arachis duranensis* and *A. ipaënsis.* The motif search was performed by MEME-suite (http://meme-suite.org/tools/meme). The scale length is represented in base pairs (bp) and the introns are rescaled to have the same length.

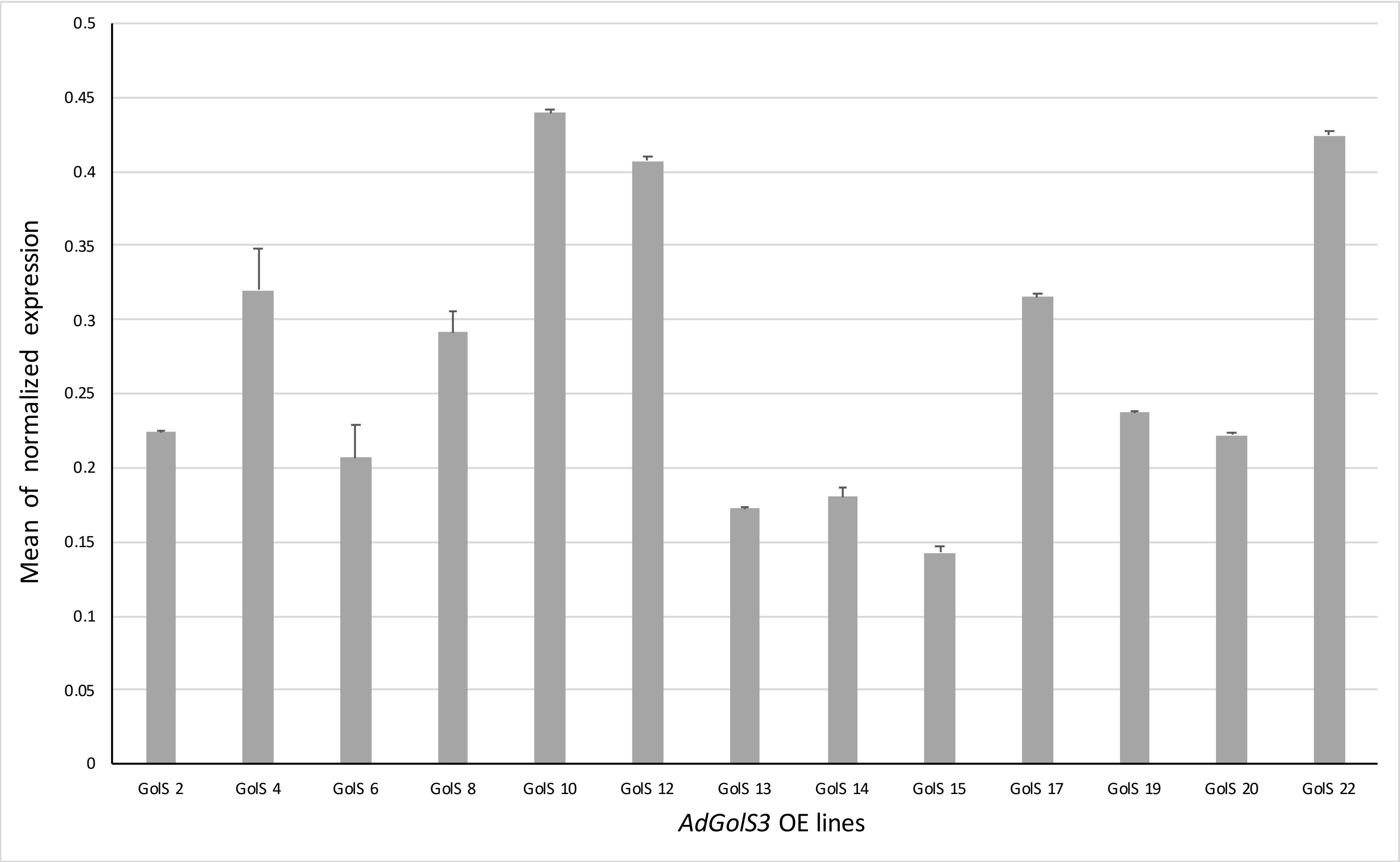


**Figure S6.** Mean of normalized expression of *AdGolS3* transgene quantified by qRT-PCR, using the *AtACT2* and *AtEF-1α* genes from *Arabidopsis* as references. Error bars are the standard errors of the means from three samples of 10 plants.

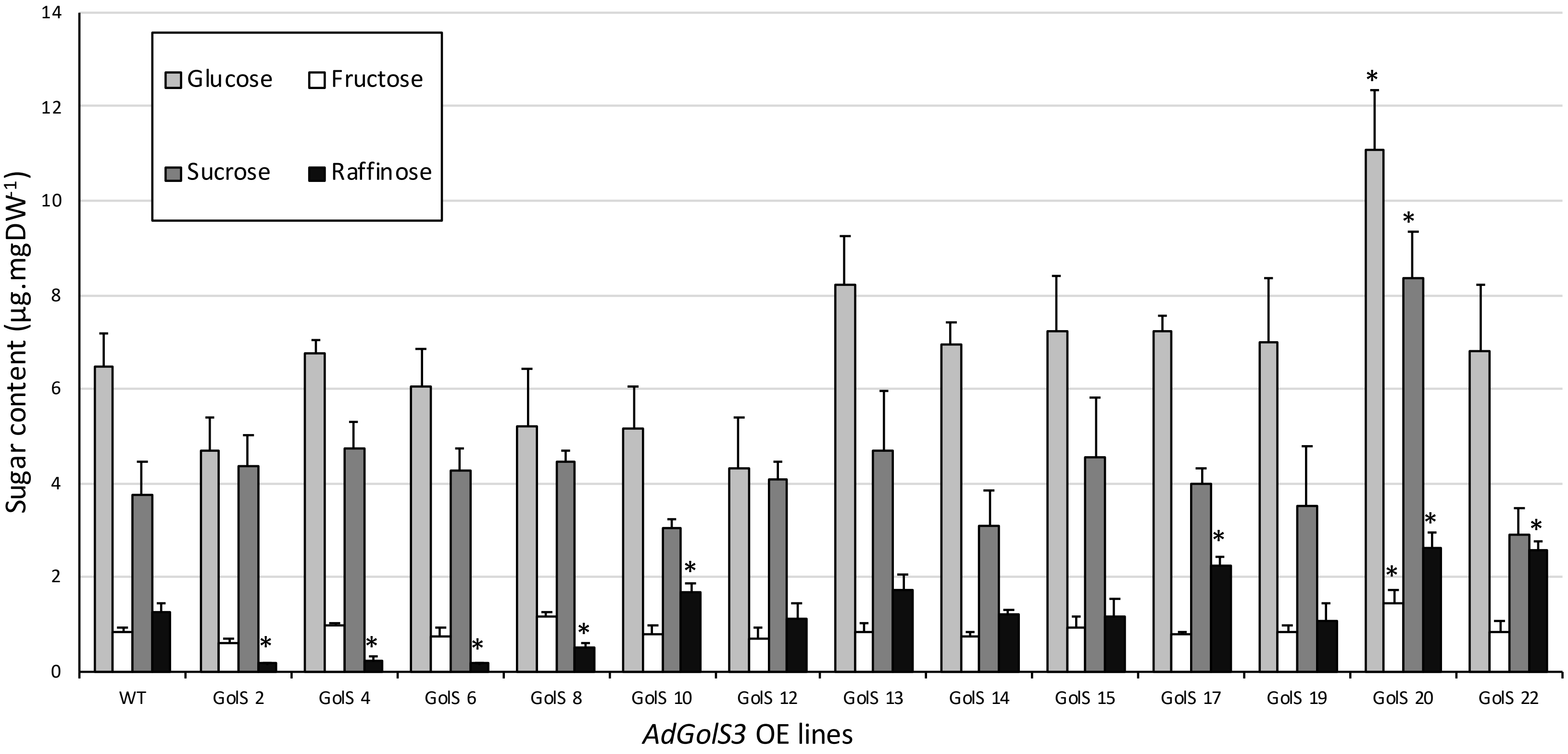


**Figure S7.** Determination of sugar content (glucose, fructose, sucrose and raffinose) in wild-type (WT) non-transgenic plants and 13 *AdGolS3* OE lines. Bars represent means and standard error. Asterisks indicate statistically significant differences for each sugar compared to WT plants (t-test; n=5; $p < 0.05$).

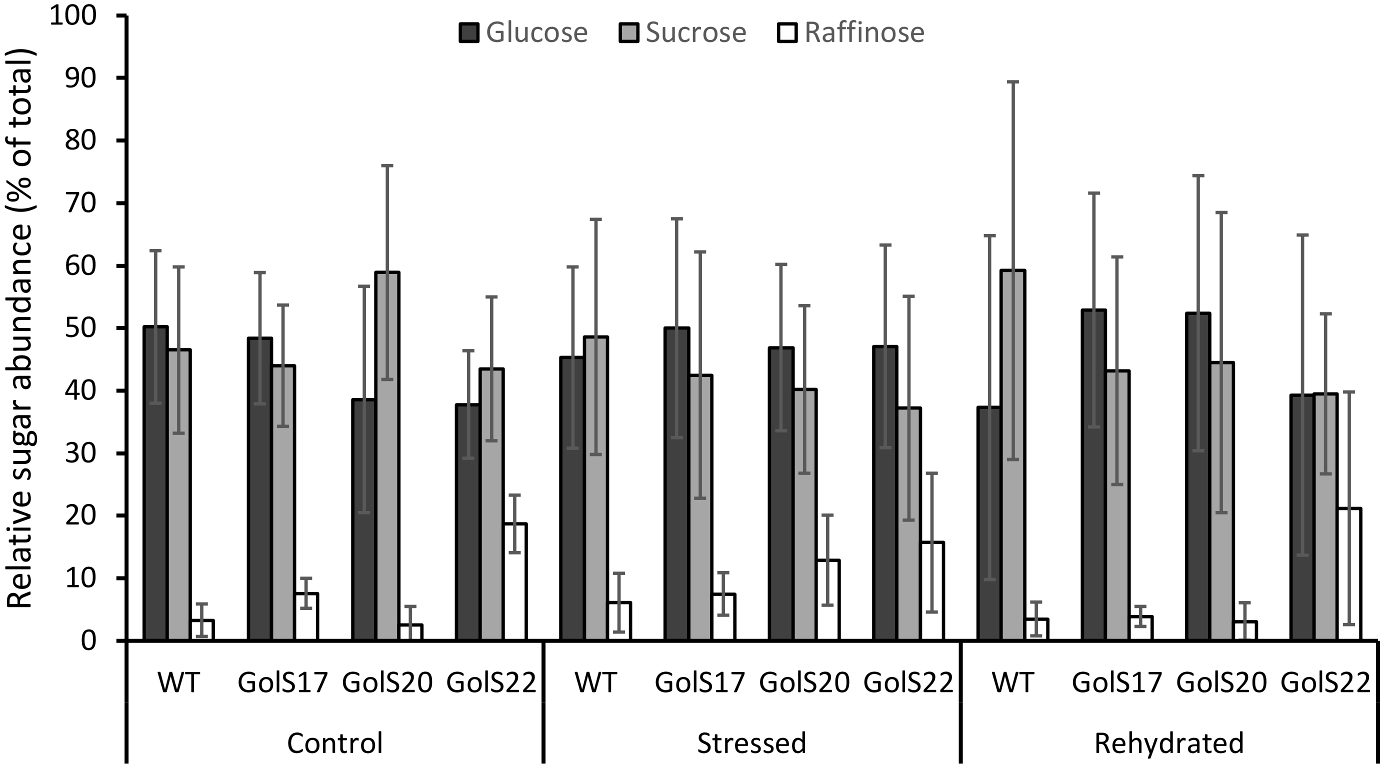


**Figure S8**: Relative percentage abundance of soluble sugars (glucose, sucrose and raffinose) extracted from leaves of wild-type (WT) untransformed plants and three OE lines (GolS17, GolS20 and GolS22) under control (CTR), stressed (STR) and rehydrated (REH) conditions. Values indicate the abundance (w/w) of each sugar as a percentage of total quantified sugar [average (AVG) and standard deviation (SD) of three to five individuals]. * indicates significant differences from WT plants for a given treatment, whilst + indicates significant differences from the control treatment for a given genotype (WT and each OE line) (t-test; $p<0.05$).

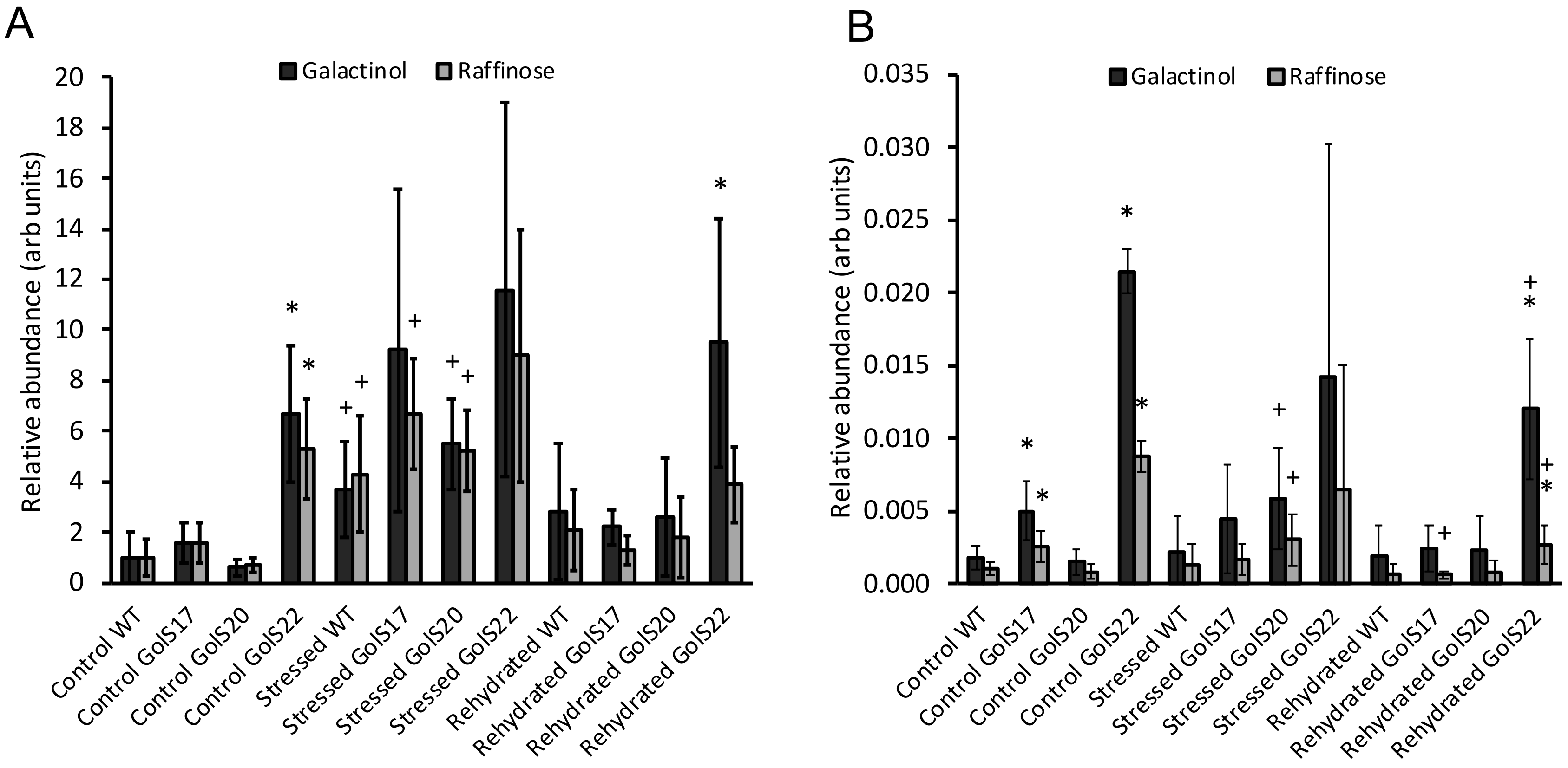


**Figure S9:** Relative abundance of galactinol and raffinose extracted from leaves of wild-type (WT) untransformed plants and three OE lines (GolS17, GolS20 and GolS22) under control, stressed and rehydrated conditions. (A) Abundance of each metabolite relative to that detected in WT plants under control conditions. (B) Abundance of each metabolite relative to the sum of all metabolites. Values are average (AVG) and standard deviation (SD) of four to five individuals and are calculated from the specific area ion count for each metabolite. * indicates significant differences from WT plants for a given treatment, whilst + indicates significant differences from the control treatment for a given genotype (WT or each OE line) (t-test, p<0.05).

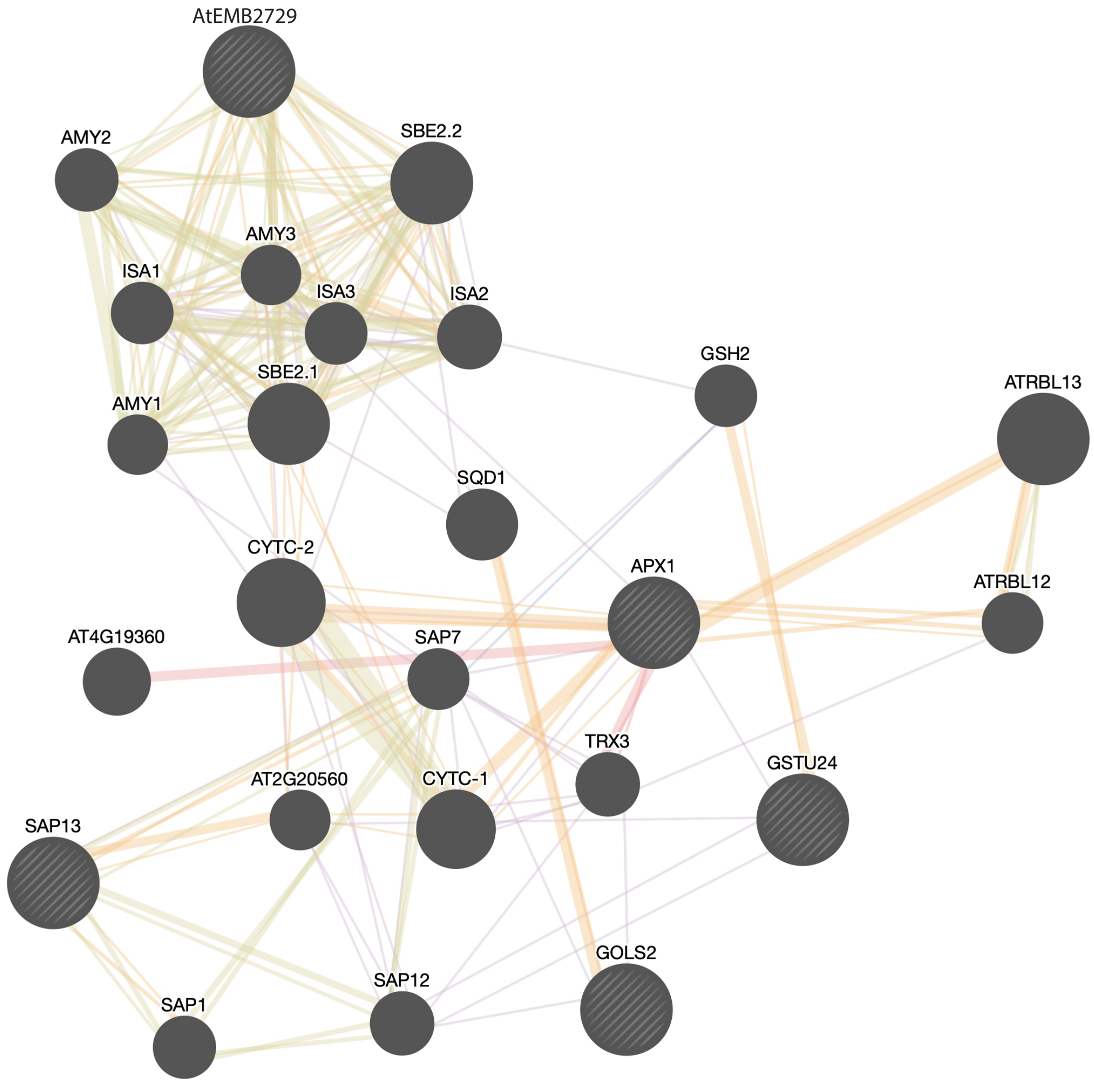


**Figure S10.** Protein networks predicted by GeneMANIA web tool for *Arabidopsis* AtGolS2. Hatched circles represent the selected coding genes (AtEMB2729, APX1, SAP13, Gols2, GSTU24). The different color nodes represent the different types of interaction.

## REFERENCES


1. Katoh, K., Misawa, K., Kuma, K. & Miyata, T. MAFFT: a novel method for rapid multiple sequence alignment based on fast Fourier transform. *Nucleic Acids Res.* **30,** 3059–3066 (2002).
2. Capella-Gutiérrez, S., Silla-Martínez, J. M. & Gabaldón, T. trimAl: a tool for automated alignment trimming in large-scale phylogenetic analyses. *Bioinformatics* **25,** 1972–1973 (2009).
3. Mota, A. P. Z. *et al.* Contrasting effects of wild Arachis dehydrin under abiotic and biotic stresses. *Front. Plant Sci.* **10,** 1–16 (2019).
4. Wang, Y. *et al.* MCScanX: a toolkit for detection and evolutionary analysis of gene synteny and collinearity. *Nucleic Acids Res.* **40,** e49--e49 (2012).
5. Endo, M., Shimizu, H., Nohales, M. A., Araki, T. & Kay, S. A. Tissue-specific clocks in *Arabidopsis* show asymmetric coupling. *Nature* **515,** 419 (2014).
6. Vinson, C. C. *et al.* Early responses to dehydration in contrasting wild *Arachis* species. *PLoS One* **13,** (2018).
7. Abuqamar, S., Ajeb, S., Sham, A., Enan, M. R. & Iratni, R. A mutation in the expansin-like A 2 gene enhances resistance to necrotrophic fungi and hypersensitivity to abiotic stress in *Arabidopsis thaliana*. *Mol. Plant Pathol.* **14,** 813–827 (2013).
8. Dixit, A. *et al.* A stress-associated protein, AtSAP13, from *Arabidopsis thaliana* provides tolerance to multiple abiotic stresses. *Plant. Cell Environ.* **41,** 1171–1185 (2018).
9. Irani, S. & Todd, C. D. Exogenous allantoin increases *Arabidopsis* seedlings tolerance to NaCl stress and regulates expression of oxidative stress response genes. *J. Plant Physiol.* **221,** 43–50 (2018).
10. Czechowski, T., Stitt, M., Altmann, T., Udvardi, M. K. & Scheible, W.-R. Genome-wide identification and testing of superior reference genes for transcript normalization in *Arabidopsis*. *Plant Physiol.* **139,** 5–17 (2005).
